# Stress system concordance as a predictor of longitudinal patterns of resilience in adolescence

**DOI:** 10.1017/S0954579423000731

**Published:** 2023-07-12

**Authors:** Andrea Wiglesworth, Jessica Butts, Katherine A. Carosella, Salahudeen Mirza, Victoria Papke, Jason José Bendezú, Bonnie Klimes-Dougan, Kathryn R. Cullen

**Affiliations:** 1 Psychology, University of Minnesota Twin Cities, Minneapolis, MN, USA; 2 Public Health, Division of Biostatistics, University of Minnesota Twin Cities, Minneapolis, MN, USA; 3 Psychology, The Pennsylvania State University, University Park, PA, USA; 4 Psychiatry and Behavioral Sciences, University of Minnesota Twin Cities, Minneapolis, MN, USA

**Keywords:** resilience, stress response, adolescence, depressive symptoms, self-worth

## Abstract

Resilience promotes positive adaptation to challenges and may facilitate recovery for adolescents experiencing psychopathology. This work examined concordance across the experience, expression, and physiological response to stress as a protective factor that may predict longitudinal patterns of psychopathology and well-being that mark resilience. Adolescents aged 14–17 at recruitment (oversampled for histories of non-suicidal self-injury; NSSI) were part of a three-wave (T1, T2, T3) longitudinal study. Multi-trajectory modeling produced four distinct profiles of stress experience, expression, and physiology at T1 (High-High-High, Low-Low-Low, High-Low-Moderate, and High-High-Low, respectively). Linear mixed-effect regressions modeled whether the profiles predicted depressive symptoms, suicide ideation, NSSI engagement, positive affect, satisfaction with life, and self-worth over time. Broadly, concordant stress response profiles (Low-Low-Low, High-High-High) were associated with resilient-like patterns of psychopathology and well-being over time. Adolescents with a concordant High-High-High stress response profile showed a trend of greater reduction in depressive symptoms (*B* = 0.71, *p* = 0.052), as well as increased global self-worth (*B* = −0.88, *p* = 0.055), from T2 to T3 compared to the discordant High-High-Low profile. Concordance across multi-level stress responses may be protective and promote future resilience, whereas blunted physiological responses in the presence of high perceived and expressed stress may indicate poorer outcomes over time.

## Introduction

Resilience is a dynamic process that promotes positive adaptation to threatening circumstances (Cicchetti, 2013; Curtis & Cicchetti, 2003). Resilience mechanisms contribute to the variability in outcomes after exposure to a stressor and are implicated in protection against the development of psychopathology in particular (Cicchetti, 2010). While studies on resilience have largely focused on recovery from a traumatic event, and experiences of maltreatment in particular (Masten et al., 1990), we propose that resilience can also be considered as a process that promotes recovery from psychopathology to restore a thriving state. Adolescent-onset psychopathology is associated with antecedent adversities, is a developmental phenomenon that requires adaptation, and is prospectively linked to poorer mental health, educational, occupational, and social outcomes that persist into early adulthood (Davies et al., 2018). However, resilience processes may promote adaptive functioning and eventual recovery among a subset of individuals affected by psychopathology in adolescence. To summarize, though definitions of resilience typically call for identification of a discrete stressor, we argue that the multifactorial and cumulative stresses associated with adolescent-onset psychopathology can be situated in the resilience framework as “major assaults on the developmental process” (Luthar et al., 2000). Therefore, highlighting protective mechanisms which promote healthy adaptation for psychopathology-affected adolescents is critical to ameliorating downstream sequelae and functional impairment.

Depressive symptoms and self-injurious thoughts and behaviors commonly emerge in adolescence and produce substantial distress for youth. Major depressive disorder has a past-year prevalence of 16% in adolescence (Daly, 2022). Self-injurious thoughts and behaviors, while co-occurring with depression, are distinct transdiagnostic constructs and include suicidal thoughts and behaviors (e.g., suicide ideation and suicide attempt) as well as non-suicidal self-injury (NSSI). Suicide ideation and attempt have a lifetime prevalence of 12 and 4% respectively in adolescence (Nock et al., 2013). NSSI, which is the engagement in self-injury *without* intent to die, has an adolescent lifetime prevalence of 17% (Swannell, Martin, Page, Hasking, & St John, 2014). NSSI is often conceptualized as a coping tool, as it typically occurs in the context of severe negative emotions, such as those associated with depressive disorders and suicide ideation, and serves to manage stressors and remove negative affect in the present (Başgöze, Wiglesworth, Carosella, Klimes-Dougan, & Cullen, 2021; Bentley et al., 2014; Lloyd-Richardson et al., 2007). However, NSSI can be life-threatening and is associated with future suicide attempts (Castellví et al., 2017; Nock et al., 2008; Ribeiro et al., 2016). Thus, adolescents who engage in NSSI represent a group of young people who are experiencing significant distress and are experimenting with different ways to navigate these difficult experiences.

Despite the stressors associated with adolescent psychopathology, some youth appear to recover from these symptoms across the course of adolescence. Extant longitudinal research suggests that, while some adolescents with major depressive disorder continue experiencing recurrent depressive episodes and suicidal thoughts and behaviors well into adulthood, a substantial proportion recover (Curry et al., 2011; Johnson et al., 2018; Weissman et al., 1999). Though the literature is more limited on NSSI recovery, many adolescents who engage in NSSI appear to desist in late adolescence or early adulthood (Plener et al., 2015; Rajhvajn Bulat et al., 2023). These patterns of recovery implicate the presence of resilience processes and protective mechanisms, which, when understood, could be leveraged to restore adaptive functioning in youth suffering from psychopathology.

In addition to reductions in symptoms of psychopathology, evidence of increased well-being over time, especially in the wake of stressors and difficulties, is another important marker of resilience. Importantly, well-being and psychopathology do not represent separate ends of a single dimension; rather, they represent distinct– albeit often inversely related– domains that individuals can experience together in varying combinations (de Vries et al., 2021). Well-being has been conceptualized as the combination of feeling good and functioning effectively, with the ability to manage negative emotions to a certain extent (Ruggeri et al., 2020). Thus, it is unsurprising that well-being and resilience are strongly linked (de Vries et al., 2021; Klainin-Yobas et al., 2021; Li & Hasson, 2020). Life satisfaction, self-worth and positive affect are critical domains of well-being that are linked with resilience (Cohn et al., 2009; Liu et al., 2012). However, well-being is not typically a focus of research on depression and self-injury in adolescence, which tends to narrowly measure “deficits” in functioning. In this regard, understanding the resilience processes underlying the positive modulation of these aspects of well-being, even when baseline circumstances may be distressing, could provide greater insight to adaptation in the context of stress.

Conceptual models hold that resilience processes involve transactions across systems and levels of analysis which move individuals towards healthy adaptation (Masten et al., 2021). In the current study, we focus on a multi-level illustration of the adolescent stress response as a potential resilience mechanism involved in developmental patterns towards adaptation and away from maladaptation. One of these levels-of-analysis involves the hypothalamic-pituitary adrenal (HPA) axis, which orchestrates an array of energy-mobilizing processes in response to stress (Herman et al., 2005). One of the major end products of the HPA axis pathways is cortisol, an adrenocorticoid steroid hormone which modulates gene expression across tissues including the brain (Godoy et al., 2018). Heightened patterns of HPA functioning (both basal functioning and response to stressors as indexed by the steroid hormone cortisol) have been repeatedly demonstrated in those with depression (Iob et al., 2020; Juruena et al., 2018; Klimes-Dougan et al., 2019; Lopez-Duran et al., 2009), though these largely cross-sectional studies are unable to provide insights into the dynamic interaction between depressive symptom recovery and baseline HPA axis characteristics. In contrast to the patterns associated with depression, adolescents with NSSI demonstrate a flattening of the HPA response to social stressors (Kaess et al., 2012; Klimes-Dougan et al., 2019), a pattern which appears to be related to greater NSSI severity (Başgöze et al., 2021). Evidence for both heightened and lowered HPA activity in depression and self-injurious thoughts and behavior may reflect developmental progression and adaptation to chronic stress at the biological level of analysis. That is, the HPA system is calibrated for negative feedback, such that consistently high levels of cortisol inhibit further HPA axis activation and subsequent hypercortisolemia. For example, in the context of chronic stress, allostatic changes can recalibrate the HPA axis to a hyporeactive physiological setpoint. While this adaptation is advantageous in that it mitigates potential negative sequelae of sustained glucocorticoid-mediated signaling pathways (e.g., neurotoxicity), it is also accompanied by certain trade-offs (Ellis & Giudice, 2014; Rao & Androulakis, 2019). Dampened responses to stressors may leave individuals unequipped to deal with future stressful situations (Leistner & Menke, 2020; Taborsky et al., 2021). As these disparate patterns of HPA activity have been observed in adolescents with depression and NSSI, two highly comorbid clinical phenomena, it is plausible that there are multiple patterns of HPA activity linked to risk and resilience processes. These patterns may be better understood when following reports of symptoms prospectively over time. Indeed, capturing fluctuations in indicators of psychopathology and well-being across time, as opposed to at a single assessment, provides better insight to the nature and stability of patterns, as resilience is dynamic and may fluctuate to some degree (Luthar et al., 2000; Wright & Masten, 2005).

Stress perception and response selection are two additional levels of analysis germane to a more comprehensive understanding of adolescent stress response functioning and, thus, resilience mechanisms. In line with the multi-level resilience framework, considering multiple domains of stress responding may be critical to arriving at a more nuanced, comprehensive understanding of developmental patterns of psychopathology symptom recovery and well-being. Correspondence across the stress system is thought to aid in the efficacious management of stressors, through orienting to the stressor and mobilizing resources for responding to the stressor (Mauss & Robinson, 2009). That is, patterns of concordance across domains of stress responses (e.g., perceived stress, behavior under stress conditions, cortisol response to stress) may indicate a well-orchestrated and flexible stress response, where the degree of hormonal response is marshaled to be commensurate with the perceived level of stress and demands to engage in goal-directed behavior in a given setting. Person-centered analytic approaches (Masten & Barnes, 2018; Von Eye & Bergman, 2003) are well-positioned for examining such patterns of the stress response and identifying profiles that may be protective.

Demonstrating the utility of a multi-level, person-centered approach, multi-trajectory modeling has been employed to characterize patterns of concordance across self-reported experience of stress, experimenter-rated expressions of stress, and physiological (cortisol) responses to stress (i.e., *stress experience-expression-physiology concordance*). As a person-centered data analytic approach, multi-trajectory modeling (Nagin et al., 2018) identifies subgroups of individuals based on shared profiles of similar intraindividual variation on growth (i.e., trajectories) across multiple indices of the phenomena of interest. In two studies, multi-trajectory modeling was applied to adolescent stress experience, expression, and physiology indices in response to a laboratory-based stressor paradigm: the Trier Social Stress Test (TSST; Kirschbaum et al., 1993). Interestingly, these studies identified similar profiles and patterns of association with psychopathology in two different sample populations. The first study included adolescents with and without depression (Bendezú, Thai, et al., 2022). In this sample, three concordant profiles of stress experience, expression, and physiology were identified: High experience, High expression, and High physiology $\left(H_{\mathrm{experi}-}H_{\mathrm{expres}-}H_{\mathrm{physio}}\right)$, Moderate experience, Moderate expression, and Moderate physiology $\left(M_{\mathrm{experi}-}M_{\mathrm{expres}-}M_{\mathrm{physio}}\right)$, and Low experience, Low expression, and Low physiology $\left(L_{\mathrm{experi}-}L_{\mathrm{expres}-}L_{\mathrm{physio}}\right)$. One discordant profile was found: High experience, High Expression, and Low Physiology $\left(H_{\mathrm{experi}–}H_{\mathrm{expres}–}L_{\mathrm{physio}}\right)$. Compared to the discordant profile, the concordant $L_{\mathrm{experi}-}L_{\mathrm{expres}-}L_{\mathrm{physio}}$ and $M_{\mathrm{experi}–}M_{\mathrm{expres}–}M_{\mathrm{physio}}$ profiles had less severe depressive symptoms, suicide ideation, and NSSI engagement. While those with the $H_{\mathrm{experi}–}H_{\mathrm{expres}–}H_{\mathrm{physio}}$ profile reported similarly severe depressive symptoms to those with the $H_{\mathrm{experi}–}H_{\mathrm{expres}–}L_{\mathrm{physio}}$ profile, they reported lower severity suicide ideation and NSSI engagement, suggesting the potential protective nature of concordant stress responding.

The second study largely replicated these findings in a group of adolescents assigned female sex at birth that was oversampled for a history of NSSI (e.g., *n* = 36 with no NSSI history, *n* = 73 with NSSI history ranging from mild to severe; Carosella, Wiglesworth, et al., Accepted for Publication). In this study, three of the four previously identified profiles were found; $L_{\mathrm{experi}–}L_{\mathrm{expres}–}L_{\mathrm{physio}}$, $H_{\mathrm{experi}–}H_{\mathrm{expres}–}H_{\mathrm{physio}}$, and $H_{\mathrm{experi}–}H_{\mathrm{expres}–}L_{\mathrm{physio}}$. While the $M_{\mathrm{experi}–}M_{\mathrm{expres}–}M_{\mathrm{physio}}$ profile was not found, a different fourth group was identified: High Experience–Low Expression–Moderate Physiology $\left(H_{\mathrm{experi}–}L_{\mathrm{expres}–}M_{\mathrm{physio}}\right)$. Again, when compared to those in the $H_{\mathrm{experi}–}H_{\mathrm{expres}–}L_{\mathrm{physio}}$ profile, those in the $L_{\mathrm{experi}–}L_{\mathrm{expres}–}L_{\mathrm{physio}}$ profile demonstrated less psychopathology including depressive symptoms and suicidal thoughts and behaviors. In this second study, however, there were not significant differences found between the $H_{\mathrm{experi}–}H_{\mathrm{expres}–}L_{\mathrm{physio}}$ and $H_{\mathrm{experi}–}H_{\mathrm{expres}–}H_{\mathrm{physio}}$ profiles in depressive symptoms or suicide ideation. Yet, those in the $H_{\mathrm{experi}–}H_{\mathrm{expres}–}H_{\mathrm{physio}}$ profile were less likely than those in the $H_{\mathrm{experi}–}H_{\mathrm{expres}–}L_{\mathrm{physio}}$ profile to report past suicide attempts. In this study, the reports of those with $H_{\mathrm{experi}–}L_{\mathrm{expres}–}M_{\mathrm{physio}}$ profiles largely mirrored those with $L_{\mathrm{experi}–}L_{\mathrm{expres}–}L_{\mathrm{physio}}$ profiles. Notably, no differences were found between profiles in the likelihood or severity of lifetime NSSI engagement. Though these cross-sectional studies provide greater insight into person-specific stress response profiles and the possible role of stress system concordance in resilience processes, an opportunity remains to examine the potential protective nature of stress system concordance in the context of longitudinal patterns of psychopathology and well-being.

Situated in a resilience framework, this paper expands on our prior cross-sectional work with adolescents who were oversampled for a history of NSSI (Carosella, Wiglesworth, et al., Accepted for Publication) by examining the association between the previously identified profiles of stress experience-expression-physiology concordance and developmental patterns of psychopathology and well-being across three timepoints. As there were no differences between the four identified profiles in the proportion of those with versus without a history NSSI, nor in the severity of NSSI history, a fundamental assumption of this study is that youth across all four profiles have experienced stressors requiring adaptation. For the purposes of this work, patterns of persistently low or decreasing psychopathology, including depressive symptoms, suicide ideation, and NSSI, and patterns of persistently high or increasing well-being, including positive affect, satisfaction with life, and self-worth, were viewed as being indicative of resilience (e.g., signaling adaptation to stressors associated with the adolescent transition and baseline/antecedent experiences related to psychopathology). Based on the extant theory of concordance and limited literature, we hypothesized that concordant baseline stress experience-expression-physiology profiles ($L_{\mathrm{experi}–}L_{\mathrm{expres}–}L_{\mathrm{physio}}$and $H_{\mathrm{experi}–}H_{\mathrm{expres}–}H_{\mathrm{physio}}$) would be protective factors that confer resilience, as indicated by lesser psychopathology and greater well-being over time.

## Method

### Study procedures

All study procedures were approved by the Institutional Review Board at the University of Minnesota. Participants are from the BRain Imaging Development in Girls’ Emotion and Self (BRIDGES) Study, a longitudinal study that recruited adolescents assigned female sex at birth with varying histories of NSSI (Başgöze et al., 2021). Data collection consisted of three waves (T1, T2, and T3). Three visits were conducted during each time point, which entailed clinical assessments administered via semi-structured interviews as well as self-report questionnaires (for T1, after completing the informed consent process with the parent or guardian and assent with the adolescent), a modified Trier Social Stress Test (TSST), and neuroimaging procedures. The primary goal of the BRIDGES Study was to use a Research Domain Criteria (RDoC) approach to examine sustained threat, cognitive control and self-processing in adolescents who were oversampled for engagement in NSSI.

Inclusion criteria for the BRIDGES Study were being assigned female sex at birth and between the ages of 12 and 17. The study sample was also oversampled for those with current or past engagement in NSSI, as we aimed to include adolescents with a range from no to severe NSSI engagement. Exclusion criteria for this study included lifetime substance use disorder (with an exception for tobacco use disorder) at T1, bipolar disorder diagnosis at T1, intellectual or developmental disability, major medical illness that would impact their neurobiology, and magnetic resonance imaging (MRI) contraindications at T1. Additional study information has been published elsewhere (Basgöze et al., 2021).

### Measures

#### Descriptives

##### Demographics.

Parents and guardians reported on participant demographics, including race and ethnicity at T1 and age (coded with two decimal points following the year) and family income at all three time points. For this study, family income was dichotomized prior to inclusion as a covariate in the longitudinal models, though the disaggregated data are reported in Table 1. Income was coded categorically to indicate income < $60,000 versus ≥ $60,000 (the approximate median income for the surrounding area in 2019 at the midpoint of data collection).

##### Clinical Diagnoses.

The Kiddie Schedule of Affective Disorders and Schizophrenia Present and Lifetime Version for DSM-5 (KSADS-5; Kaufman et al., 1997) is a semi-structured interview that was independently conducted with adolescents and the parent or guardian. This measure demonstrates excellent test-retest reliability and high interrater agreement. Adolescent and parent/guardian interviewers conducted a consensus process based on symptom reports to assign DSM-5 psychiatric diagnoses, which are used to characterize our sample herein.

##### Medications.

Parents reported on child medication use at T1, which were included in the study as this time point coincides with the TSST administration and cortisol data collection. Medications were coded as a dichotomous variable to indicate whether the parent reported a medication that is suggested to impact the HPA axis and assessment of cortisol via saliva collection (Granger et al., 2009).

#### Psychopathology outcomes of interest

##### Depressive Symptoms.

The Beck Depression Inventory II (BDI-II; Beck et al., 1996; Beck et al., 1996) is a 21-item scale that measures depressive symptoms over the prior two week period. Participants responded to each item on a four-point scale, and all items were summed for a total score with higher scores indicating more severe symptoms. This measure shows good internal consistency in past research α = 0.91. The BDI-II also showed excellent internal consistency in this sample (Table S1).

##### Suicide Ideation.

The Beck Scale for Suicidal Ideation (BSSI; Beck et al., 1979) is a 21-item scale that measures severity of suicidal thoughts, preparatory behaviors, and attempts. Participants indicate their responses on a three-point scale, and total scores were calculated as a sum of all items with higher scores indicating more severe suicide ideation. The BSSI computerized self-report version demonstrates excellent internal consistency (Cronbach α = 0.96). Past research has demonstrated the utility of sum scores of either the first five or first 19 items (de Beurs, Fokkema, de Groot, de Keijser, & Kerkhof, 2015). For the present study, the sum score of the first five items was used as the outcome of interest, as items six through 19 were not administered to those who denied symptoms in items four and five. The internal consistency of the five-item BSSI in this sample was good in the current sample (Table S1).

##### Nonsuicidal Self-Injury.

The Self-Injurious Thoughts and Behaviors Interview (SITBI; Nock et al., 2007) is a semi-structured interview used to assess participant engagement in NSSI. For descriptive purposes in this study, we examined lifetime number of NSSI episodes and severity of injury from the T1 assessment to create four groups: No NSSI, Mild NSSI (fewer than four past episodes involving significant tissue damage, or unlimited NSSI episodes with no tissue damage), Moderate NSSI (four or more past NSSI episodes occurring less than once per month with significant tissue damage), and Severe NSSI (four or more past NSSI episodes occurring more than once per month with significant tissue damage). Prior work from our group has also used this approach, which resembles approaches taken by others (e.g., Esposito-Smythers et al., 2010; Muehlenkamp et al., 2017), to identify group differences in mechanisms of sustained threat (Başgöze et al., 2021). For our longitudinal analyses, we examined past year counts of NSSI engagement, which were log transformed to approximate a more normalized distribution (Table S1), as our outcome of interest.

#### Well-being outcomes of interest

##### Positive Affect.

The Positive and Negative Affect Schedule Expanded Form (PANAS-X; Clark & Watson, 1994) is a 60-item self-report measure of 13 specific affective states. Participants indicate their responses on a five-point scale, and the subscale scores are calculated as a sum of constituent items. The Positive Affect subscale (10 items) score was retained, and higher scores indicate higher levels of positive affect. The Positive Affect subscale demonstrated good to excellent internal consistency (Table S1).

##### Satisfaction with Life.

The Satisfaction with Life Scale (SLS; Diener et al., 1985) is a five-item self-report measure of an individual’s evaluative judgment of their overall satisfaction with life, independent of the affective component. Participants indicate their responses on a seven-point scale, and total scores are calculated as a sum of all items with higher scores indicating greater life satisfaction. The internal consistency of the SLS in this sample was excellent (Table S1).

##### Self-worth.

The Self-Perception Profile for Adolescents (SPPA; Harter, 1988) is a 45-item (nine subscales) self-report measure of adolescent self-concept. Within each domain, participants indicate their responses on a four-point scale of competence. The global self-worth subscale was retained for this study (higher scores indicate higher levels of self-worth) and showed good to excellent internal consistency in this sample (Table S1).

#### Trier social stress test (Stress experience, expression, and physiology)

This study examined data from the T1 modified TSST. During the modified TSST (Kirschbaum et al., 1993; Klimes-Dougan et al., 2014; Yim et al., 2010), participants were instructed to prepare a five minute speech that they might give if they were to introduce themselves to a new class. They then delivered their speech to two trained examiners. After the five minute speech, the adolescent performed verbal arithmetic calculations for five minutes. Throughout the speech and math tasks, the examiners maintained neutral expressions and refrained from providing reassurance or positive feedback. The correlations between the metrics of stress experience, expression, and physiology during TSST speech and math tasks can be found in Table S2.

Following the completion of the speech and math tasks, participants rated their *experience of stress* during the speech preparation, speech task, math task, and post-test period (e.g., “right now”) on a five-point scale, where 1 was the lowest stress and 5 was the highest.

During the TSST, each examiner independently rated their observations of the participants’ *expression of stress* during the speech task and math task on a six-point scale where 1 = “Not stressed at all,” 2 = “A little stressed,” 3 = “Moderately stressed,” 4 = “Very stressed,” 5 = “Considered discontinuing the procedure because they looked so stressed,” and 6 = “Discontinued the procedure because they were showing signs of distress.” Raters were instructed to conduct ratings based on signs of stress expression including fidgeting, blushing, freezing, abrupt pauses, and verbal references to stress. The independent ratings were averaged to produce a separate mean score for each task. Examiner ratings were moderately correlated for each task ($r_{\mathrm{speech}}=0.64$, $r_{\mathrm{math}}=0.72$, p’s<0.001).

Salivary cortisol output in response to the TSST served as an index of participant *physiological response to stress*. This procedure included five saliva samples, collected pre-task (“CORT 1”), post-speech and math task at + 15 minutes from CORT1 (“CORT2”), +30 minutes (“CORT3”), +45 minutes (“CORT4”), and + 60 minutes (“CORT5”). Participants were instructed to push saliva through a straw into a vial. Vials were stored on site in a −25°C freezer and then shipped to Universitat Trier in Trier, Germany for assay with methods consistent to (Dressendörfer et al., 1992). Cortisol values that were greater than three standard deviations of the mean were winsorized prior to analysis.

### Data analysis

#### Sample Characteristics.

Descriptive statistics including bivariate Pearson’s and Spearman’s correlations were performed on the demographic and clinical variables to characterize the sample and nature of the data. Outcomes of interest were all linear and were checked for normality (e.g., skewness and kurtosis). Past year NSSI engagement was the only variable log transformed prior to analysis. Correlations and descriptives are in Tables S1 and S2.

#### Multi-trajectory Modeling.

Procedures used here are described in detail in our prior manuscript (Carosella, Wiglesworth, et al., Accepted for Publication). To identify profiles of within-person variation on stress experience, expression, and physiology, we employed multi-trajectory modeling (Nagin et al., 2018) in SAS version 9.4 using the PROC TRAJ procedure (SAS Institute, 2010) with MULTGROUPS option. The PROC TRAJ procedure utilizes Full-Information Maximum Likelihood (FIML) to estimate model parameters in the presence of missing data, a method that is most appropriate when such data are assumed to be missing completely at random (MCAR). A nonsignificant (Little, 1988) MCAR test (*X*^2^ = 72.167, *p* = 0.20) suggested that FIML was an appropriate missing data approach. For the current study, multi-trajectory modeling followed three steps: a) model specification, b) model adequacy evaluation, and c) trajectory distinction analysis. To specify the best fitting model, linear, quadratic, and cubic polynomial parameters were initially estimated for stress expression (two data points), experience (four data points), and physiology (five data points) trajectories, respectively. For each model specification step (e.g., one-profile solution, two-profile solution), non-significant polynomial parameters were removed until a solution containing only significant polynomial parameters was obtained. As recommended (Andruff et al., 2009; Helgeson et al., 2004; Louvet et al., 2009), linear polynomial parameters were retained irrespective of statistical significance.

After this solution was obtained, the log Bayes factor approximation $\left[2{\text{log}}_{\text{e}}\left({\text{B}}_{10}\right)\right]$ was examined to assess model fit, where a log Bayes factor greater than 10 is said to be strong evidence for the superior fit of the more complex model (e.g., three-profile solution) relative to the less complex model (e.g., two-profile solution) (Jones & Nagin, 2007). Given our sample size, model specification was limited to four profiles. After the best fitting model was specified, we evaluated the adequacy of the solution with three metrics: average posterior probability $\left({\text{AvePP}}_{\text{j}}\right)$, odds of correct classification $\left({\text{OCC}}_{\text{j}}\right)$, and the ratio of the probability of profile assignment to the proportion of adolescents assigned to profiles $\left({\text{Prob}}_{\text{j}}/{\text{Prop}}_{\text{j}}\right)$ (Nagin et al., 2018). An ${\text{AvePP}}_{\text{j}}$ greater than 0.70, OCC_j_ greater than 5.00, and ${\text{Prob}}_{\text{j}}/{\text{Prop}}_{\text{j}}$ close to 1.00 provide evidence of multi-trajectory model adequacy. Following adequacy evaluation, trajectory distinction analyses were conducted. Specifically, a series of Wald tests compared intercept and polynomial parameter estimates for each identified profiles’ stress, experience, expression, and physiology trajectories. The results of these analyses help to show how baseline levels and reactivity patterns for each trajectory are relatively “higher” or “lower” across the identified profiles and, thus, how trajectories across profiles were distinct from one another.

#### Longitudinal Modeling.

Sample sizes for each outcome measure at each time point are presented in Table S1. To better understand potential patterns of missing data that might inform our analyses or interpretations of our results, we conducted logistic regression analyses to determine whether T1 demographic or clinical characteristics predicted patterns of missing data across the entire sample and within each profile at T2 and T3 (see Tables S3–S8).

To model whether the baseline profiles of stress experience, expression, and physiology were associated with changes over time in outcomes of interest (i.e., depressive symptoms, suicide ideation, past year NSSI engagements, positive affect, global self-worth, satisfaction with life), we conducted a series of linear mixed-effect regression models using the *lme4* package (Bates et al., 2015) in R version 4.2.2 (R Core Team, 2022). These models account for the nested structure of the longitudinal data (e.g., observations nested within individuals). Each model included the fixed effect of profile and time point as main effects as well as their interaction. Profile was coded categorically, where each concordant stress response profile ($L_{\mathrm{experi}–}L_{\mathrm{expres}–}L_{\mathrm{physio}}$ and $H_{\mathrm{experi}–}H_{\mathrm{expres}–}H_{\mathrm{physio}}$) was entered as the reference group in separate analyses. This decision was based on our interest in understanding stress response concordance, as opposed to discordance, as a protective factor. Time point was also coded categorically and T2 was set as the reference time point, which allowed us to examine change in slope from T1 to T2 and T2 to T3. The profile by time point interaction allowed us to examine whether the slope (e.g., change in outcome) from T1 to T2 and from T2 to T3 differed between the profiles. Age (e.g., associated with size and structure of key regulatory glands; Linton & Dorshkind, 2004), income (e.g., associated with HPA hypoactivation; Evans et al., 2012; Lupien et al., 2000), and HPA acting medication use at T1 (Granger et al., 2009) were all included as covariates given their theoretical links to stress responses and outcomes of interest. The covariate of age accounted for both age and time between assessments. In cases where income was missing at a given visit, the most recent income value was used instead. Given the limited variability in race/ethnicity in our sample and the limited correlation of minoritized race/ethnicity with outcomes of interest, this variable was not included as a covariate in our models. The *emmeans* R package (Lenth, 2023) was used to produce contrasts from the fitted linear mixed-effect models to test within group changes in indexes of psychopathology and well-being over time (e.g., whether slopes differed significantly) and between-group differences at each time point. Data visualizations were produced using the *ggplot2* R package (Wickham, 2016). While we use the standard *p* = 0.05 threshold to determine whether results are statistically significant, we also report results where *p* ≤ 0.1 below given the possibility of limited statistical power to detect small to medium effects when comparing discrete groups.

## Results

### Overall sample characteristics

A total of 164 adolescents were enrolled in the BRIDGES Study. Of these youth, 113 had usable salivary cortisol data from the T1 TSST and were included in multi-trajectory modeling analyses. Finally, of those youth, four participants were missing income data across all timepoints, making our final sample of participants 109 adolescents ages 12.20–17.02 at T1. Descriptive statistics of age, race and ethnicity, and income can be found in Table 1. In the current sub-sample, based on the T1 SITBI, NSSI severity groups were as follows: No NSSI (*n* = 35); Mild NSSI (*n* = 10); Moderate NSSI (*n* = 41); Severe NSSI (*n* = 23). Those with a history of NSSI were more likely than those without to meet criteria for at least one clinical diagnosis (94.5% versus 47.1%). Current symptoms consistent with Major Depressive Disorder were the most common in our sample (53.2%) followed by Attention-deficit Hyperactivity Disorder (29.4%) and Generalized Anxiety Disorder (25.7%). Suicide ideation (62.4%), planning (39.4%), and attempts (31.2%) were also endorsed frequently by youth in our sample.

### Multi-trajectory modeling

Results from multi-trajectory model analyses in this sample are reported in extensive detail elsewhere (Carosella et al., Accepted for Publication). Relevant parameter estimates, adequacy indices, and results of our trajectory distinction analyses can be found in the supplement (Table S9). Multi-trajectory model specification arrived at increasingly complex solutions: two- to one-group comparison $\left[2{\text{log}}_{\text{e}}\left({\text{B}}_{10}\right)=263.66\right]$, three- to two-group comparison $\left[2{\text{log}}_{\text{e}}\left({\text{B}}_{10}\right)=56.88\right]$, four- to three-group comparison $\left[2{\text{log}}_{\text{e}}\left({\text{B}}_{10}\right)=10.46\right]$. Following the methods outlined in (Nagin, 2005) for model specification and model adequacy evaluation, the four-group model was selected and found to fit the data well. To characterize the resultant profiles, we relied on significantly differing aspects of the trajectories (e.g., intercepts, response patterning) to illustrate how the profiles differed from one another.

**Figure 1.**
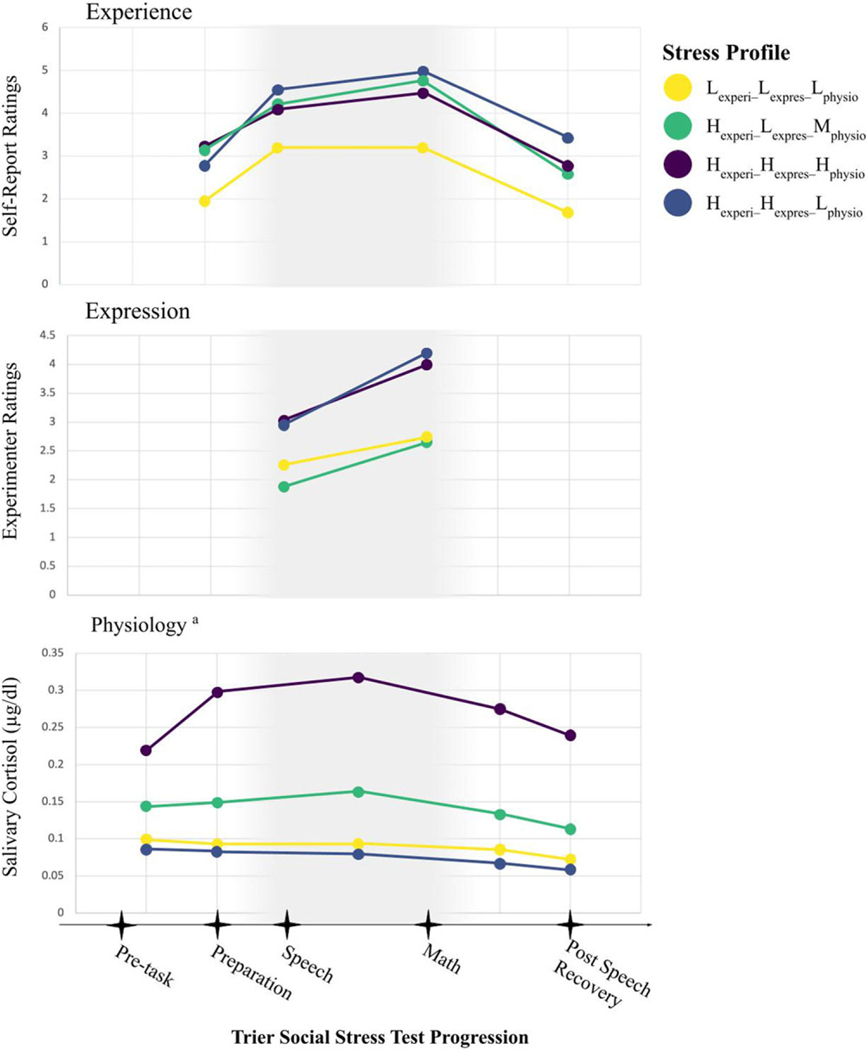
Profiles indicated in the final four-group solution from the multi-trajectory model. raw scores for experience, expression, and physiology are indicated on the y-axis; ^a^ trier social stress test event markers for physiology are estimated based on the time-lag in salivary cortisol presentation; ${\text{L}}_{\text{experi}–}{\text{L}}_{\text{expres}–}{\text{L}}_{\text{physio}}=\text{low experience}$, low expression, low physiology, ${\text{H}}_{\text{experi}–}{\text{H}}_{\text{expres}–}{\text{H}}_{\text{physio}}=\text{high experience}$, high expression, high physiology, ${\text{H}}_{\text{experi}–}{\text{L}}_{\text{expres}–}{\text{M}}_{\text{physio}}=\text{high experience}$, low expression, moderate physiology, ${\text{H}}_{\text{experi}–}{\text{H}}_{\text{expres}–}{\text{L}}_{\text{physio}}=\text{high experience}$, high expression, low physiology.

As previously discussed, the four profiles identified were characterized as $L_{\mathrm{experi}–}L_{\mathrm{expres}–}L_{\mathrm{physio}}$ (*n* = 41), $H_{\mathrm{experi}–}H_{\mathrm{expres}–}L_{\mathrm{physio}}$(*n* = 28), $H_{\mathrm{experi}–}H_{\mathrm{expres}–}H_{\mathrm{physio}}$(*n* = 19), and $H_{\mathrm{experi}–}L_{\mathrm{expres}–}M_{\mathrm{physio}}$ (*n* = 25; Fig. 1). Four individuals included in our multi-trajectory models were missing gross income data, which was a covariate in our longitudinal analyses, resulting in smaller group sizes for the $L_{\mathrm{experi}–}L_{\mathrm{expres}–}L_{\mathrm{physio}}$ (*n* = 39) and $H_{\mathrm{experi}–}H_{\mathrm{expres}–}L_{\mathrm{physio}}$ (*n* = 26) profiles in this study. No significant group differences between the profiles based on age, proportion of racially or ethnically minoritized youth, or gross family income (<$60,000 versus ≥$60,000) were evident (all *p*’*s* > 0.05). Further, as previously described, there were no significant differences between profiles in NSSI history or severity at T1 (see Table S10).

### Psychopathology predictive models

When comparing longitudinal patterns of psychopathology between the $L_{\mathrm{experi}–}L_{\mathrm{expres}–}L_{\mathrm{physio}}$ profile and the two discordant stress profiles, results indicated that adolescents with the $H_{\mathrm{experi}–}H_{\mathrm{expres}–}L_{\mathrm{physio}}$ profile demonstrated significantly higher depressive symptoms at T2 (*B* = 0.86, *p* < 0.001; Table S11). Post-hoc contrasts revealed that differences in depressive symptoms between these profiles were also significant at T1 and T3 (Table S12). However, the changes in depressive symptoms from Y1 to Y2 and from Y2 to Y3 were not statistically significant within each profile (Table S13), nor statistically different between the two profiles, indicating similarly flat patterns of depressive symptoms for the $L_{\mathrm{experi}–}L_{\mathrm{expres}–}L_{\mathrm{physio}}$ and $H_{\mathrm{experi}–}H_{\mathrm{expres}–}L_{\mathrm{physio}}$ profiles. The difference in suicide ideation severity between the $L_{\mathrm{experi}–}L_{\mathrm{expres}–}L_{\mathrm{physio}}$ and $H_{\mathrm{experi}–}H_{\mathrm{expres}–}L_{\mathrm{physio}}$ profiles was statistically significant at T1, though not statistically significant and moderately sized at T2 and T3 (Table S12). There were no significant or trend-level effects for NSSI engagement when comparing the two discordant profiles to the $L_{\mathrm{experi}–}L_{\mathrm{expres}–}L_{\mathrm{physio}}$ profile.

**Figure 2.**
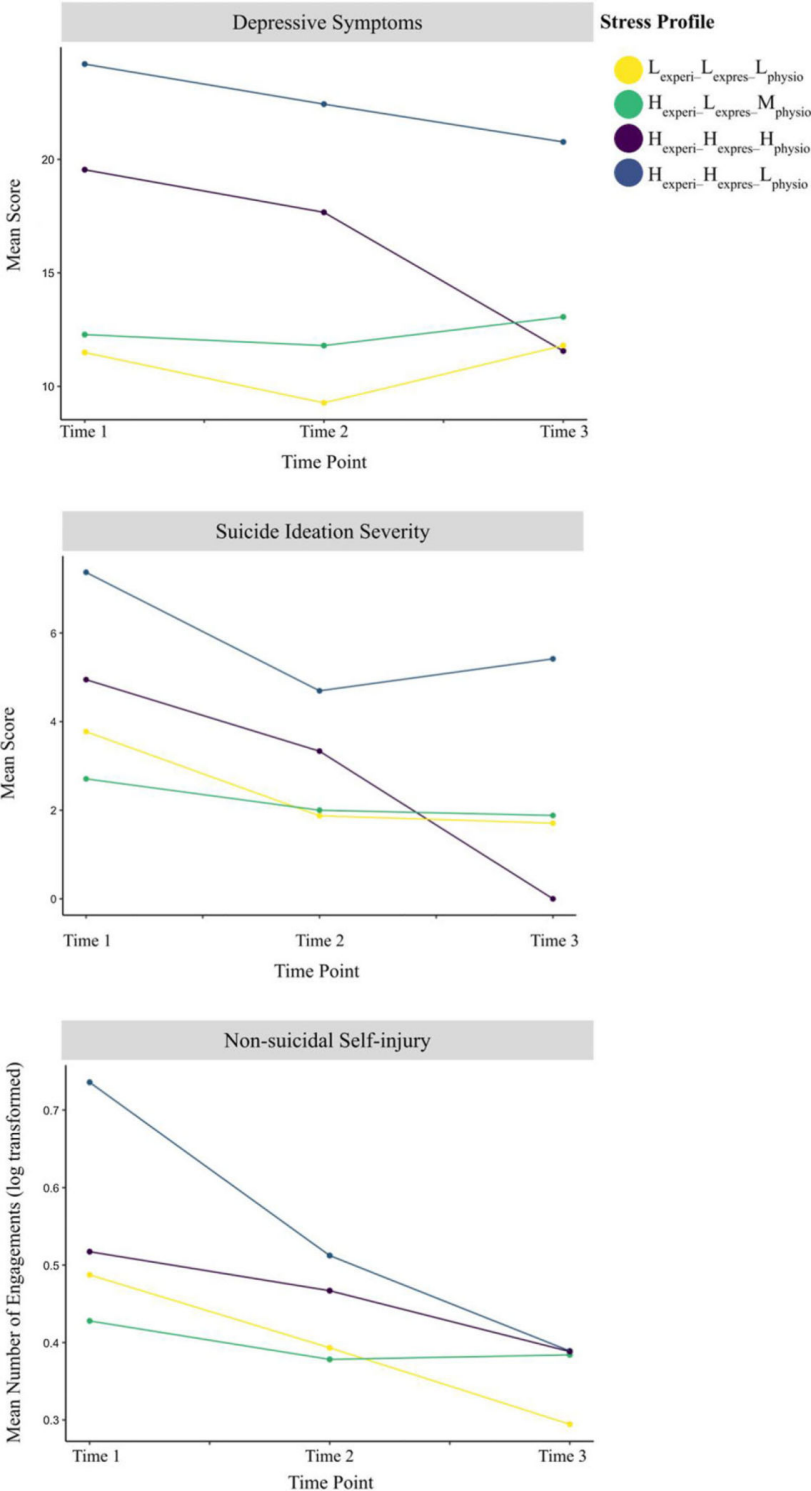
Longitudinal patterns of reporting of symptoms of psychopathology disaggregated by profile.${\text{L}}_{\text{experi}–}{\text{L}}_{\text{expres}–}{\text{L}}_{\text{physio}}=\text{low experience}$, low expression, low physiology, ${\text{H}}_{\text{experi}–}{\text{H}}_{\text{expres}–}{\text{H}}_{\text{physio}}=\text{high experience}$, high expression, high physiology, ${\text{H}}_{\text{experi}–}{\text{L}}_{\text{expres}–}{\text{M}}_{\text{physio}}=\text{high experience}$, low expression, moderate physiology, ${\text{H}}_{\text{experi}–}{\text{H}}_{\text{expres}–}{\text{L}}_{\text{physio}}=\text{high experience}$, high expression, low physiology.

Parameter estimates for longitudinal models of psychopathology where the $H_{\mathrm{experi}–}H_{\mathrm{expres}–}H_{\mathrm{physio}}$ profile is the reference group can be found in Table 2. Adolescents with the concordant $H_{\mathrm{experi}–}H_{\mathrm{expres}–}H_{\mathrm{physio}}$ profile demonstrated a trend of greater decreases in depressive symptoms from T2 to T3 as compared to those with the $H_{\mathrm{experi}–}H_{\mathrm{expres}–}L_{\mathrm{physio}}$ profile (B = 0.71, p = 0.052). Similarly, a trend emerged when comparing the $H_{\mathrm{experi}–}H_{\mathrm{expres}–}H_{\mathrm{physio}}$ profile to the $H_{\mathrm{experi}–}L_{\mathrm{expres}–}M_{\mathrm{physio}}$ profile from T2 to T3 (*B* = 0.60, *p* = 0.085). Longitudinal patterns of suicide ideation severity were similar to the depressive symptom patterns from T2 to T3 (see Fig. 2), again with a trend in the data where those with the $H_{\mathrm{experi}–}H_{\mathrm{expres}–}H_{\mathrm{physio}}$ profile reported a greater decrease in suicide ideation than did those with the $H_{\mathrm{experi}–}H_{\mathrm{expres}–}L_{\mathrm{physio}}$ profile (*p* = 0.100), though this non-significant finding may be due to limited statistical power. Post-hoc contrasts indicated that, following these trends, those with the $H_{\mathrm{experi}–}H_{\mathrm{expres}–}L_{\mathrm{physio}}$ profile reported significantly higher suicide ideation severity at T3 but not at T1 or T2 than those with the $H_{\mathrm{experi}–}H_{\mathrm{expres}–}H_{\mathrm{physio}}$ profile (Table S13). Indeed, despite having similar suicidal ideation severity scores at T1, at T3 youth with the $H_{\mathrm{experi}–}H_{\mathrm{expres}–}H_{\mathrm{physio}}$ profile reported very limited suicidal ideation, whereas the reported suicidal ideation of those with the $H_{\mathrm{experi}–}H_{\mathrm{expres}–}L_{\mathrm{physio}}$ profile were, on average, unchanged from T1 to T3. No significant interaction or main effects for time point and profile were identified when examining longitudinal patterns of past year NSSI engagements when comparing discordant profiles to the concordant $H_{\mathrm{experi}–}H_{\mathrm{expres}–}H_{\mathrm{physio}}$ profile (Table 2). Despite the lack of significant findings, the $H_{\mathrm{experi}–}H_{\mathrm{expres}–}L_{\mathrm{physio}}$ profile group demonstrate an approximate 40% decrease in mean past year NSSI engagement between T1 and T3.

### Well-being predictive models

When comparing longitudinal patterns of well-being between the $L_{\mathrm{experi}–}L_{\mathrm{expres}–}L_{\mathrm{physio}}$ profile and the two discordant stress profiles, there were several significant main effects (Table S14). Compared to adolescents with the $L_{\mathrm{experi}–}L_{\mathrm{expres}–}L_{\mathrm{physio}}$ profile, those with the $H_{\mathrm{experi}–}H_{\mathrm{expres}–}L_{\mathrm{physio}}$ profile demonstrated significantly lower global self-worth (*B* = −0.68, *p* = 0.018) and satisfaction with life (*B* = −0.66, *p* = 0.017) at T2; global self-worth was also significantly lower for those with the $H_{\mathrm{experi}–}H_{\mathrm{expres}–}L_{\mathrm{physio}}$ profile at T1 and T3 (Table S15). At T2, those with the $H_{\mathrm{experi}–}L_{\mathrm{expres}–}M_{\mathrm{physio}}$ profile also showed significantly lower global self-worth compared to those with the $L_{\mathrm{experi}–}L_{\mathrm{expres}–}L_{\mathrm{physio}}$ profile (*B* = −0.70, *p* = 0.015). Similar to the psychopathology models, the changes in well-being indexes from T1 to T2 and from T2 to T3 within each discordant profile were not significantly different from the changes over time in the $L_{\mathrm{experi}–}L_{\mathrm{expres}–}L_{\mathrm{physio}}$ profile, indicating similar longitudinal patterns of well-being across these groups (Table S14, S16).

**Figure 3.**
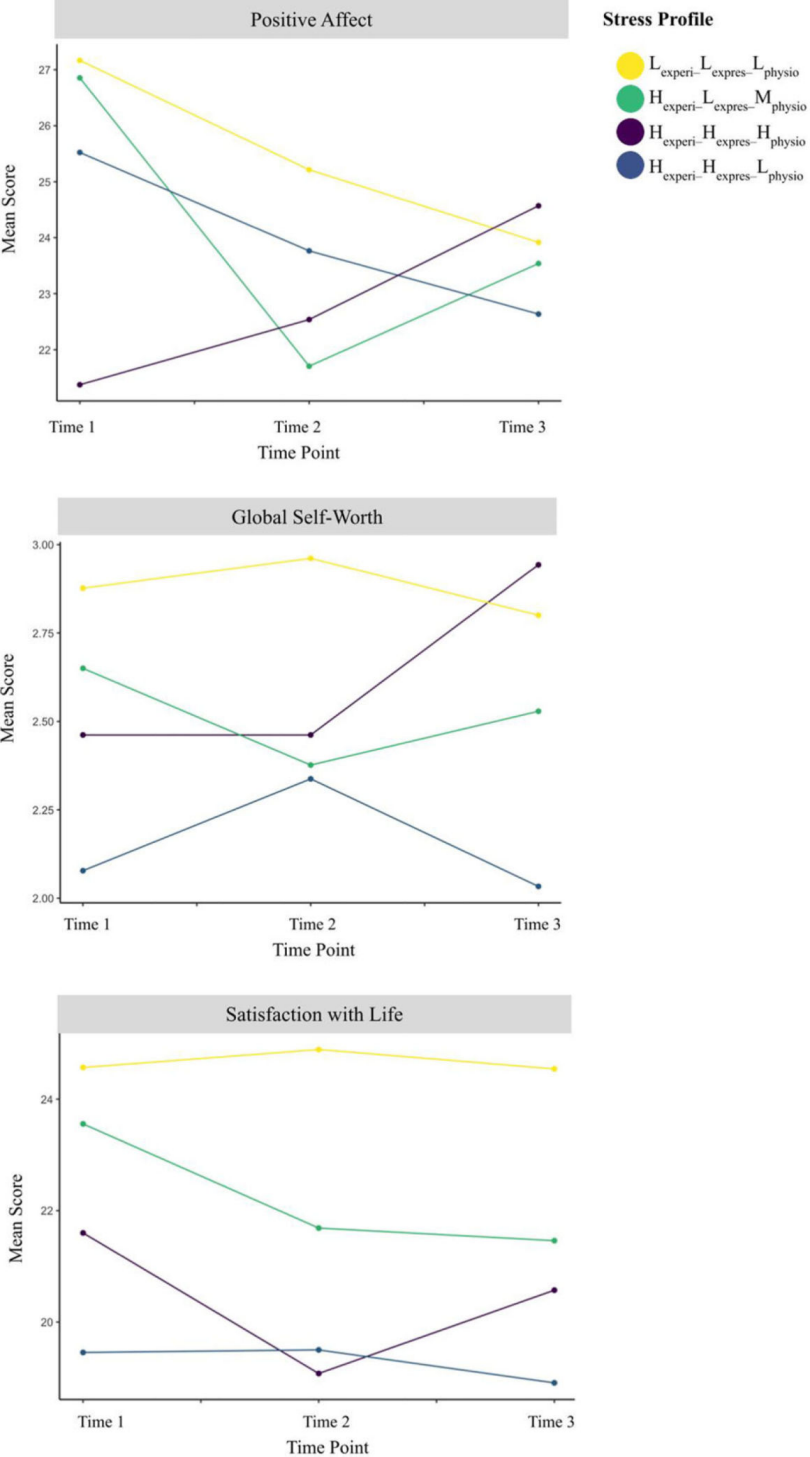
Longitudinal patterns of reporting of symptoms of well-being disaggregated by profile.${\text{L}}_{\text{experi}–}{\text{L}}_{\text{expres}–}{\text{L}}_{\text{physio}}=\text{low experience}$, low expression, low physiology, ${\text{H}}_{\text{experi}–}{\text{H}}_{\text{expres}–}{\text{H}}_{\text{physio}}=\text{high experience}$, high expression, high physiology, ${\text{H}}_{\text{experi}–}{\text{L}}_{\text{expres}–}{\text{M}}_{\text{physio}}=\text{high experience}$, low expression, moderate physiology, ${\text{H}}_{\text{experi}–}{\text{H}}_{\text{expres}–}{\text{L}}_{\text{physio}}=\text{high experience}$, high expression, low physiology.

Parameter estimates for longitudinal models of well-being where the $H_{\mathrm{experi}–}H_{\mathrm{expres}–}H_{\mathrm{physio}}$ profile is set as the reference group are found in Table 3. Results from longitudinal models of positive affect revealed a significant profile by time point interaction where those with the concordant $H_{\mathrm{experi}–}H_{\mathrm{expres}–}H_{\mathrm{physio}}$ profile demonstrated greater increases in positive affect from T1 to T2 as compared to the discordant $H_{\mathrm{experi}–}L_{\mathrm{expres}–}M_{\mathrm{physio}}$ profile (*B* = 1.00, *p* = 0.009), which demonstrated a decreasing trend in positive affect over this time period. Although differences in positive affect from T1 to T2 and T2 to T3 between the between the $H_{\mathrm{experi}–}H_{\mathrm{expres}–}H_{\mathrm{physio}}$ and $H_{\mathrm{experi}–}H_{\mathrm{expres}–}L_{\mathrm{physio}}$ profiles were not statistically significant (*p* = 0.096 and *p* = 0.191 respectively), visual inspection illustrates that those in the $H_{\mathrm{experi}–}H_{\mathrm{expres}–}H_{\mathrm{physio}}$ profile showed an upward trend in positive affect over time that was not observed for those in other profiles (Figure 3). Similarly, when examining global self-worth, the concordant $H_{\mathrm{experi}–}H_{\mathrm{expres}–}H_{\mathrm{physio}}$ profile demonstrated a marginally greater increase in self-worth from T2 to T3 as compared to the $H_{\mathrm{experi}–}H_{\mathrm{expres}–}L_{\mathrm{physio}}$ profile (*B* = −0.88, *p* = 0.055). The $H_{\mathrm{experi}–}H_{\mathrm{expres}–}L_{\mathrm{physio}}$ profile demonstrated a trend of decreasing self-worth over that same time period, resulting in significantly different reports of self-worth at T3 (Table S15). There were no notable trends when comparing profiles on satisfaction with life, as these scores largely did not change over time (Figure 3).

## Discussion

This study examined whether profiles of multimodal stress response, defined using a person-centered approach, predicted patterns of resilience (as indicated by trends in psychopathology and well-being over time) in adolescents with a range of histories of NSSI engagement. Key strengths of the study included the longitudinal design, the focus on a high-risk sample of adolescents with high rates of NSSI and psychopathology histories, and the person-centered, multidimensional characterization of stress response trajectories that did not rely solely on self-report data. While three of the profiles demonstrated broadly resilient patterns indicating improvement in well-being and diminishing psychopathological symptoms over time, the $H_{\mathrm{experi}–}H_{\mathrm{expres}–}L_{\mathrm{physio}}$ profile showed the poorest outcomes with respect to longitudinal trends, including tempered patterns of recovery in depression and suicide ideation severity, and blunted positive affect and self-worth over time. However, it is notable that this profile showed the greatest reduction of NSSI engagement over time, though these slopes were non-significant.

Much of the adolescent resilience literature focuses on vulnerability to developing psychopathology using high-risk samples, where “high-risk” is often as defined by varied stress exposures and “resilience” is manifested as an absence of psychopathology. However, in keeping with recent calls for research that move away from an absence-of-deficit approach to defining resilience (Fergus & Zimmerman, 2005; Masten et al., 2021; Yates et al., 2015), the current work posits that resilience mechanisms can continue to operate even after the development of symptoms of psychopathology. In other words, those who experience psychopathology and/or struggle with suicidal thoughts and behaviors and NSSI may still draw upon protective mechanisms, which in turn promote recovery. Thus, we examined a sample of adolescents who, at the onset of the study, were already experiencing high rates of psychopathology including suicidal thoughts and behaviors and self-injury. By examining whether concordant stress response profiles predicted longitudinal patterns of psychopathology and well-being indicative of resilience, we uncovered patterns that generate hypotheses about potential protective mechanisms facilitating recovery in adolescence.

Here we focused on the stress response system as a potential key protective factor in influencing resilience patterns in adolescence. In particular, based on theory of stress correspondence and our own past work (Carosella et al., Accepted for Publication), we hypothesized that concordance across the experience, expression, and physiological levels of the stress response would confer resilience. As hypothesized, adolescents with the two concordant profiles showed the most consistent patterns of resilience, with $H_{\mathrm{experi}–}H_{\mathrm{expres}–}H_{\mathrm{physio}}$ profiles showing largely consistent trends of recovery (e.g., an approximate 50% decrease in depressive symptoms on average from T1 to T3), and adolescents with $L_{\mathrm{experi}–}L_{\mathrm{expres}–}L_{\mathrm{physio}}$ profiles showing maintained patterns of relatively low psychopathology and high well-being. In contrast, adolescents in the $H_{\mathrm{experi}–}L_{\mathrm{expres}–}M_{\mathrm{physio}}$ profile showed less consistent outcomes, with relatively low psychopathology but some fluctuations (though not statistically significant) in positive affect over time. Noteably, similar to previous cross-sectional findings (Bendezú et al., 2022), those in the $H_{\mathrm{experi}–}H_{\mathrm{expres}–}L_{\mathrm{physio}}$ profile demonstrated a non-resilient pattern in which depressive symptoms and suicide ideation severity largely persisted over time, and indexes of well-being remained low or trended downward.

Comparing longitudinal patterns of symptomatology between those with the $H_{\mathrm{experi}–}H_{\mathrm{expres}–}L_{\mathrm{physio}}$ and the $H_{\mathrm{experi}–}H_{\mathrm{expres}–}H_{\mathrm{physio}}$ profiles provides compelling evidence that, although the allostatic change of recalibration of the HPA system in the context of chronic stress may be adaptive in the short-term, this pattern may also have long-term consequences. That is, this pattern where the perception of threat is high but is not accompanied by a commensurately high physiological response may reflect a broader process of allostatic loading which incurs risk for delayed or dampened recovery from psychopathology and impaired growth in well-being. Similarly, another clear contrast emerges when comparing the $L_{\mathrm{experi}–}L_{\mathrm{expres}–}L_{\mathrm{physio}}$ and $H_{\mathrm{experi}–}H_{\mathrm{expres}–}L_{\mathrm{physio}}$ profiles, both of which are characterized by low physiological response to stress. However, adolescents with the $L_{\mathrm{experi}–}L_{\mathrm{expres}–}L_{\mathrm{physio}}$ profile demonstrated a stress response that was commensurately low with their low perceived and exhibited stress levels, which may reflect that they did not experience the TSST to be particularly stressful, rather than reflecting allostatic change. Accordingly, differences between these profiles in symptoms of psychopathology and well-being between were striking; results indicated that the longitudinal patterns of psychopathology and well-being were similarly flat (e.g., limited change year-to-year) for these two groups, but that for the $L_{\mathrm{experi}–}L_{\mathrm{expres}–}L_{\mathrm{physio}}$ profile this flat patterning indicated persistently lower psychopathology and higher well-being, and for the $H_{\mathrm{experi}–}H_{\mathrm{expres}–}L_{\mathrm{physio}}$ profile this flat patterning indicated persistently high psychopathology and low well-being. For example, the $H_{\mathrm{experi}–}H_{\mathrm{expres}–}L_{\mathrm{physio}}$ demonstrated depressive symptoms severity suicidal ideation severity scores about two times higher than those of the $L_{\mathrm{experi}–}L_{\mathrm{expres}–}L_{\mathrm{physio}}$ profile across the three timepoints. Thus, these results suggest that a low physiological response to stress may only confer resilience when it is consistent with a lower experience and expression of threat. Importantly, those with $L_{\mathrm{experi}–}L_{\mathrm{expres}–}L_{\mathrm{physio}}$ response profiles showed arguably the most optimal longitudinal outcomes, though adolescents with this profile were not less likely than their peers to have some history of NSSI (e.g., participants recruited based on having no history of NSSI at T1 were not overrepresented in the group characterized by this profile of stress response). Thus, this concordant profile was a predictor of favorable trends in psychopathology and well-being despite the likely presence of stressors associated with adolescent NSSI engagement.

The patterns that emerged when comparing adolescents with the $L_{\mathrm{experi}–}L_{\mathrm{expres}–}L_{\mathrm{physio}}$ and $H_{\mathrm{experi}–}H_{\mathrm{expres}–}L_{\mathrm{physio}}$ profiles on longitudinal trends of suicide ideation and NSSI are consistent with findings from a prior person-centered, multi-level study that similarly investigated a sample of female adolescents at risk for self-injurious thoughts and behaviors (Bendezú, Calhoun, Patterson, et al., 2022). In that study, youth who experienced more pronounced negative affect responses and less pronounced salivary cortisol responses to the TSST were more likely to report suicide ideation, suicide attempt, and NSSI at three-to-nine-month follow-up relative to their counterparts who experienced less pronounced negative affect and less pronounced cortisol responses. Notably, female youth in the high negative affect/low salivary cortisol and low negative affect/low salivary cortisol profiles also exhibited the lowest and highest basal positive affect levels, respectively, in the sample, which is consistent with conceptualizations of adolescent risk for self-injurious thoughts and behaviors as involving disturbance of positive valence systems (Craske et al., 2016; Hankin, 2012). Though these studies differ with respect to indices utilized to extract the profiles (e.g., emotion experiences vs. stress expression and experience), this partial overlap in profiles identified as well as similar longitudinal links to self-injurious thoughts and behaviors suggests that person-centered, multi-level approaches (such as multi-trajectory modeling) to the study of adolescent stress response functioning may reliably identify meaningful subgroups of female adolescents with varied levels of risk for psychopathology.

While the current study is built upon the hypothesis that concordance in stress processing may facilitate resilience processes and predict positive future outcomes, it is important to acknowledge the nuances unveiled in these results. For example, examining satisfaction with life results suggested that, while those in the $H_{\mathrm{experi}–}H_{\mathrm{expres}–}L_{\mathrm{physio}}$ profile showed the lowest and flattest pattern over time with scores that reflect slight dissatisfaction with life (e.g., score < 20; Diener et al., 1985), those in the $H_{\mathrm{experi}–}H_{\mathrm{expres}–}H_{\mathrm{physio}}$ profile also showed relatively lower satisfaction with life compared to their peers. It is possible that in this context, while the concordance at the physiological level may be protective, the high psychological burden of stress still interferes with life satisfaction. To highlight further nuances, while the $H_{\mathrm{experi}–}H_{\mathrm{expres}–}H_{\mathrm{physio}}$ profile showed consistent improvements generally in symptoms of psychopathology and well-being, adolescents in the discordant $H_{\mathrm{experi}–}L_{\mathrm{expres}–}M_{\mathrm{physio}}$ profile maintained relatively low psychopathology across the three time points, suggesting that this profile may also be protective, despite its discordant nature. The only exception to this interpretation, however, is the somewhat unstable reports of positive affect which vary (non-significantly) over time. Thus, the ways that stress response patterns map on to resilience may not simply be explained by the bifurcation of concordance and discordance.

Moreover, the potential protective nature of stress response concordance does not seem to apply indiscriminately to all outcomes, even highly related outcomes, of psychopathology and well-being. Indeed, depression, suicide ideation, and NSSI are highly related constructs that have some unique correlates and possibly unique mechanisms (Başgöze, Wiglesworth, et al., 2021). While the patterns of depression and suicide ideation were similar when comparing groups, those for NSSI engagement were less similar (e.g., adolescents in all profiles except for $L_{\mathrm{experi}–}L_{\mathrm{expres}–}L_{\mathrm{physio}}$ reporting largely similar past year NSSI engagement at T3). Further, while adolescents in the $H_{\mathrm{experi}–}H_{\mathrm{expres}–}H_{\mathrm{physio}}$ profile demonstrated somewhat consistent trends in decreasing psychopathology over time and increasing positive affect and self-worth, this patterning did not map on qualitatively to their patterns of satisfaction with life, which trended downward from T1 to T2 and upward from T2 to T3, resulting in no significant change over the course of the study. Ultimately, it is our hope that this study acts as a catalyst for hypothesis generation regarding mechanisms that facilitate resilience among those already experiencing psychopathology during adolescence.

### Limitations and future directions

While our study had many strengths in the design, there are limitations that are important to acknowledge and be pursued as future directions for this line of work. First, considerations of internal validity must be noted when interpreting the present results. While it is critical to capture the perceptions of adolescents to assess outcomes over time, and the measures used to evaluate psychopathology and well-being were primarily well-validated indexes, the observed longitudinal patterns of psychopathology and well-being are all subject to biases associated with self-report measures, and in some cases limited to count variables as an index of severity (e.g., NSSI) or have restricted variability (e.g., BSSI, SLS). Further, while diminished or low levels of psychopathology and evidence of high or increased well-being are reasonable ways to capture resilience processes, future work may benefit from conceptualizing resilience as adaptation or competence across a larger variety of domains (Luthar et al., 2000).

Our study posits that the stress response profiles may serve as a protective factor that promotes coping or successful adaptation to stressful events. A recent meta-analysis concluded that the TSST is a useful tool for eliciting a stress response in youth (Seddon et al., 2020). Yet, there remains uncertainty around the interpretation of a low cortisol response to a moderate stressor such as the TSST. Indeed, it is possible that for some who have high distress tolerance, this paradigm was not sufficient to elicit a stress response (e.g., possibly explaining the $L_{\mathrm{experi}–}L_{\mathrm{expres}–}L_{\mathrm{physio}}$ response profile). For others, the TSST may have elicited responses across other biological systems (e.g., neural stress response, sympathomedullary pathway [SAM]) which may be asymmetrical to HPA axis responding (e.g., Başgöze, Mirza, et al., 2021; Pham et al., 2023); such patterns could be related to allostatic overload and HPA recalibration (e.g., a hypothetical explanatory factor for the $H_{\mathrm{experi}–}H_{\mathrm{expres}–}L_{\mathrm{physio}}$ response profile). Additionally, however, the assessment of cortisol can be impacted by many factors such as time of day, phase of the menstrual cycle, and diet. While the study methods took care to consider these factors and, when possible, control for them, there is still room for potential error. In addition to more thoroughly accounting for these factors in the identification of stress response profiles, a future line of inquiry would be to evaluate how stress response profiles themselves may change over time as a potentially dynamic indicator of resilience. While this kind of fine-grained longitudinal data on dynamic change in stress response together with dynamic change of psychopathology will be difficult to collect, it will be necessary to resolve ongoing questions in the field.

Second, this study has limitations regarding external validity and generalizability related to the sample size and make up. Our sample size at baseline was modest. However, participant retention was a challenge (particularly given that data collection occurred amid the COVID-19 pandemic), and the available data at follow-ups was more limited. The diminished sample size at T2 and T3 presents the possibility that our study was underpowered to detect small to medium effect sizes when comparing discrete groups (e.g., profiles) in the analyses undertaken. Additionally, the study sample had limited diversity with respect to race, ethnicity, sex, gender, and socioeconomic status, limiting generalizability. Interpersonal and systemic racism, homophobia, and transphobia as well as culturally and community bound protective factors are inextricable from stress processes, psychopathology, and resilience. However, our small sample sizes of participants from different minoritized racial or ethnic backgrounds as well as gender identities and sexual orientations meant it was not possible to disaggregate these identities at the broadest levels in our models. Moreover, we did not directly measure experiences of discrimination (e.g., interpersonal and systemic) that may contribute to group differences between minoritized youth and majority youth with regard to stress profiles or resilience patterns. Given these limitations in measurement and sampling, race/ethnicity, gender identity, and sexual orientation were not included as covariates in our models. Future work will benefit from examining these questions about stress responding and resilience in racially and ethnically diverse samples or in within-group research designs and moving beyond using race as a proxy for racism and discrimination, instead measuring these factors directly (Azibo, 1988; Lett, Asabor, Beltrán, Cannon, & Arah, 2022; Needham et al., 2022). Similarly, in recruiting diverse samples with respect to gender identity and sexual orientation, the field can continue to understand how minority stress that permeates lesbian, gay, bisexual, transgender, queer, and expansive (LGBTQ+) experiences relate to these processes of interest (e.g., Hatzenbuehler & McLaughlin, 2014). Notably, all participants in this sample were assigned female sex at birth. Given that internalizing pathology and stress responses typically differ between female and male adolescents, it is likely that these results do not fully generalize to male adolescents (Mazurka et al., 2018). Finally, the proportion of the population especially likely to enroll in this research study may have involved families who, due to various forms of privilege (e.g., resources available to find a provider), were more likely to be attentive to their adolescents’ psychopathology or receptive to intervention. Thus, there may be factors related to the sample that self-select for such a study that also relate to resilience patterns.

In the current study, we posited that the adolescent period is accompanied by a number of stressors, both indexed and perpetuated by adolescent-onset psychopathology. Thus, by oversampling for those with NSSI, we expect that our study sample was enriched for adolescent stress experiences. However, there are several external factors not addressed in this study, including *de facto* measures of stress exposure prior to baseline and across follow-up, each of which frame interesting and worthwhile threads for future research on stress responses and resilience. Antecedent experiences of severe maltreatment, as one example, likely contribute to physiological and neurobiological stress response systems and psychopathology over time (Hosseini-Kamkar et al., 2021; McLaughlin et al., 2019; Schär, Mürner-Lavanchy, Schmidt, Koenig, & Kaess, 2022). Moreover, adaptive physiological stress processing is bolstered by experiences within parent-child dyads, families, and neighborhood contexts (Gunnar & Donzella, 2002). For example, parental emotion socialization may be an antecedent experience that can bolster advantageous responses to stress, potentially leading to less severe symptomatology and more positive adaptation over time (White et al., 2021). In this naturalistic longitudinal study, involvement in treatment was not included as an exclusion criterion as part of our study procedures. Instead, our study team frequently provided recommendations for treatment when adolescents were identified to need a higher level of care than was presently being provided to them. However, it is unknown whether involvement in treatment, due to or separate from recommendations provided by our team clinicians, impacted youths’ psychopathology and well-being. As a final example, the potential stressors incurred from the COVID-19 pandemic, and their impacts on longitudinal patterns of psychopathology and well-being, were not addressed in this study. While all participants in our study sample completed the T1 assessment including the TSST prior to the onset of the pandemic, many adolescents completed the T2 and T3 time points at various stages during the pandemic. Thus, adolescents’ stress response profiles may have had an effect on how youth navigated pandemic related stressors, *and* pandemic related stressors may have played an important role in modulating adolescents’ patterns of psychopathology and well-being over time (Carosella et al., 2021; Carosella, Mirza, Başgöze, Cullen, & Klimes-Dougan, 2023).

Continued work investigating multi-level markers of adaptive processes is critical to advancing a process-informed understanding of resilience (Cicchetti, 2010). In particular, expanding the repertoire of physiological and neurobiological measures will continue to aid in uncovering the multifactorial processes underlying protection and recovery. This work focused specifically on the HPA axis as a key biological component of the response to threat, although other mechanisms including those which may be assessed by neuroimaging are promising next steps. Recent work by our group suggested that stress system concordance was associated with greater positive frontolimbic connectivity (Bendezú, Thai, Wiglesworth, Cullen, & Klimes-Dougan, 2022). Further, we hope that the present study can spur future hypotheses about the role of concordance across the levels of the stress response system as it pertains to resilience. Linking molecular markers (e.g., peripheral hormones, oxidative stress mechanisms, gene expression, epigenetic modification) to brain and behavior could clarify coactions and bidirectional influences.

### Implications for intervention

Our study contributes to a burgeoning literature highlighting the promise of multiple-levels-of-analysis, person-centered approaches in characterizing risk and resilience processes (Cicchetti & Dawson, 2002; Cicchetti & Rogosch, 1996), with implications for the evaluation of interventions designed to move biological rhythms implicated as resilience mechanisms. For example, awareness of the utility of multi-system approaches (e.g., analyzing HPA function in tandem with peripheral systems) in distinguishing well-regulated versus dysregulated biological function has begun to increase (Bendezú, Calhoun, et al., 2022; Buss et al., 2018; Chen et al., 2020). However, biologically potent interventions have overwhelmingly focused on a single biomarker, notably cortisol (Dozier, Roben, Caron, Hoye, & Bernard, 2018; Fisher et al., 2016). Restricting analysis to single systems (e.g., HPA axis in isolation) may therefore limit understanding unique patterns of comprehensive psychobiological dysregulation (e.g., experience-expression-physiology stress response concordance) that may be more or less amenable to change resulting from intervention, precluding full understanding of an intervention’s efficacy. Indeed, while some studies show evidence of HPA recalibration in the context of recovery from depressive symptoms (Aihara et al., 2007; Yuuki et al., 2005), others suggest that HPA responses remain altered even following symptom remission (Bhagwagar et al., 2003; Lange et al., 2013). Given that studies now show experience-expression-physiology stress response concordance to be linked to psychopathology and well-being both concurrently and longitudinally, one logical next step might be to utilize person-centered, multi-level approaches to study whether youth with discordant stress response profiles (e.g., $H_{\mathrm{experi}–}H_{\mathrm{expres}–}L_{\mathrm{physio}}$) recalibrate towards stress response concordance (e.g., $L_{\mathrm{experi}–}L_{\mathrm{expres}–}L_{\mathrm{physio}}$) following intervention for depression and related sequelae (e.g., suicide ideation, NSSI) and whether such recalibration is linked to symptom reduction at follow-up. Indeed, a recent study employing a multiple-levels-of-analysis, person-centered design showed recalibration of dysregulated HPA-SAM stress response asymmetry (e.g., High HPA-Low SAM) towards well-regulated HPA-SAM stress response symmetry (e.g., Low HPA-Low SAM) as a function of assignment to psychosocial intervention (Bendezú & Wadsworth, 2023). Whether similar findings emerge when experience-expression-physiology stress response concordance is the mechanism of risk and therapeutic action remains to be seen.

Moreover, the present findings provide an opportunity to identify individuals which may be in the most need of treatment due to their risk for persistent patterns of psychopathology and low well-being. The results suggest that individuals demonstrating a discordant response to threat (e.g., $H_{\mathrm{experi}–}H_{\mathrm{expres}–}L_{\mathrm{physio}}$) may be in particular need of targeted intervention. For example, adolescents reporting high experience of stress may benefit from dialectic behavioral therapy and other approaches involving mindfulness to help them regulate and navigate intensely stressful situations. And yet, a multi-systemic approach may be necessary, including both individual-focused interventions (e.g., evidence-based, skills-focused psychotherapy) and parent-focused interventions designed to support parents and provide training in how they relate to their adolescents who are suffering from depression (e.g., Reigstad et al., 2022). Interestingly, some alterations in the environment have been accompanied by enhanced responsiveness of the HPA axis (Fisher et al., 2011). In a randomized control intervention with teachers, students in the *banking time* condition (which aimed to improve a teachers interaction quality with a particular child) showed a steeper diurnal slope at the end of the school year than children in the low and no treatment conditions (Hatfield & Williford, 2017). Similarly, institutionalized children who are adopted generally show increased diurnal cortisol, however those who show flatter diurnal patterns post-adoption demonstrate more behavioral and emotional problems (Kroupina et al., 2012). While the potential therapeutic nature of restoring stress response concordance is speculative at this juncture, process-focused approaches which facilitate the adaptive functioning of the basic stress response system do present as a compelling route to intervention (Yates et al., 2015).

## Conclusion

In addition to promoting positive adaptation that may stave off the development of psychopathology, resilience processes may aid in the recovery from psychopathology and cultivation of well-being in adolescence. This study showed that using a person-centered approach to characterize multimodal stress response profiles in at-risk adolescents was useful in understanding how concordant stress responses act as a protective factor in adolescence to predict resilient patterns of depressive symptoms and self-worth over time. This work extends past research by suggesting that these experience, expression, and physiological response profiles not only highlight concurrent risks, but are also likely to have implications for resilience by predicting changes over time in both psychopathology and well-being. Taken together, our findings help to identify possible pathways toward developing personalized interventions directed at fostering resilience and ultimately reducing the burden of distress and despair associated with adolescent-onset psychopathology. We hope that the current work will invite future research examining multiple dimensions of resilience and how these dimensions unfold over time even after the onset of adolescent psychopathology.

## Supplementary Material

1

## Figures and Tables

**Table 1.** Descriptive statistics of the study sample

	Total sample (*N* = 109)	$L_{\mathrm{experi}–}L_{\mathrm{expres}–}L_{\mathrm{physio}}$ (*n*=39)	$H_{\mathrm{experi}–}H_{\mathrm{expres}–}H_{\mathrm{physio}}$ (*n*=19)	$H_{\mathrm{experi}–}L_{\mathrm{expres}–}M_{\mathrm{physio}}$ (*n*=25)	$H_{\mathrm{experi}–}H_{\mathrm{expres}–}L_{\mathrm{physio}}$ (*n*=26)
Age, *M* (*SD*)					
Time 1	14.9 (1.2)	14.8 (1.3)	14.9 (1.1)	15.2 (1.1)	14.9 (1.2)
Time 2	16.1 (1.2)	15.9 (1.4)	16.2 (0.9)	16.3 (1.2)	16.2 (1.2)
Time 3	17.3 (1.3)	17.0 (1.3)	17.5 (1.2)	17.5 (1.3)	17.2 (1.2)
Race (non-Hispanic/Latinx), *N* (%)					
American Indian	1 (0.9)	1 (2.6)	0 (0.0)	0 (0.0)	0 (0.0)
Asian	3 (2.8)	2 (5.1)	1 (5.3)	0 (0.0)	0 (0.0)
White	81 (74.3)	29 (74.4)	12 (63.2)	18 (72.0)	22 (84.6)
Asian/White	2 (1.8)	0 (0.0)	0 (0.0)	1 (4.0)	1 (3.8)
American Indian/White	1 (0.9)	0 (0.0)	0 (0.0)	0 (0.0)	1 (3.8)
Black/White	5 (4.6)	1 (2.6)	1 (5.3)	3 (12.0)	0 (0.0)
Native Hawaiian or Pacific Islander/White	1 (0.9)	0 (0.0)	1 (5.3)	0 (0.0)	0 (0.0)
Other (not specified)/White	1 (0.9)	1 (2.6)	0 (0.0)	0 (0.0)	0 (0.0)
Multiracial (not specified)	2 (1.8)	0 (0.0)	2 (10.5)	0 (0.0)	0 (0.0)
Race (Hispanic/Latinx), *N* (%)					
White	8 (7.3)	3 (7.7)	2 (10.5)	2 (8.0)	1 (3.8)
Black/White	1 (0.9)	0 (0.0)	0 (0.0)	0 (0.0)	1 (3.8)
American Indian/Black/White	1 (0.9)	1 (2.6)	0 (0.0)	0 (0.0)	0 (0.0)
Other (not specified)	2 (1.8)	1 (2.6)	0 (0.0)	1 (4.0)	0 (0.0)
Gross annual income at Time 1, *N* (%)					
≤ $24,999	8 (7.1)	3 (7.3)	2 (10.5)	2 (8.0)	1 (3.6)
$25,000-$39,999	10 (8.8)	2 (4.9)	2 (10.5)	3 (12.0)	3 (10.7)
$40,000-$59,999	9 (8.8)	5 (12.2)	1 (5.3)	2 (8.0)	2 (7.1)
$60,000-$89,999	16 (13.3)	4 (9.8)	4 (21.1)	3 (12.0)	4 (14.3)
$90,000-$179,999	46 (40.7)	17 (41.5)	9 (47.4)	8 (32.0)	12 (42.9)
≥ $180,000	20 (17.7)	8 (19.5)	1 (5.3)	7 (28.0)	4 (14.3)
Taking HPA Acting Medications at Time 1, *N* (%)					
Yes	45 (41.3)	16 (41.0)	9 (47.4)	5 (20.0)	14 (53.8)

*Note*. $L_{\mathrm{experi}–}L_{\mathrm{expres}–}L_{\mathrm{physio}}=\text{Low experience}$, Low expression, and Low physiology, $H_{\mathrm{experi}–}L_{\mathrm{expres}–}M_{\mathrm{physio}}=\text{High experience}$, Low expression, and Moderate physiology, $H_{\mathrm{experi}–}H_{\mathrm{expres}–}H_{\mathrm{physio}}=\text{High experience}$, High expression, and High physiology, $H_{\mathrm{experi}–}H_{\mathrm{expres}–}L_{\mathrm{physio}}=\text{High experience}$, High expression, and Low physiology.

**Table 2.** Linear mixed effect regression model results demonstrating group differences in indices of psychopathology over time based on stress experience, expression, and physiology profile

	Depressive symptoms *B*, b (SE)		Suicide ideation severity *B*, b (SE)		Non-suicidal self-injury *B*, b (SE)	
Age (years)	0.15	1.861 (1.197	0.02	0.048 (0.171)	0.08	0.049 (0.058)
Takes Medication Affecting HPA	0.22**	5.684 (2.053)	0.23**	0.937 (0.287)	−0.03	0.154 (0.098)
Above Median Income	−0.10	−2.948 (1.897)	−0.10	−0.458 (0.304)	0.13	−0.043 (0.099)
Time Point (Reference: Time 2)						
Time 1	0.20	2.533 (3.193)	0.34	0.666 (0.645)	0.12	0.068 (0.185)
Time 3	−0.66*	−8.183 (3.668)	−0.62	−1.219 (0.760)	−0.24	−0.140 (0.204)
Profile (Reference:$H_{\mathrm{experi}–}H_{\mathrm{expres}–}H_{\mathrm{physio}}$)						
$H_{\mathrm{experi}–}H_{\mathrm{expres}–}L_{\mathrm{physio}}$	0.29	3.606 (3.941)	0.19	0.368 (0.663)	0.01	0.005 (0.202)
$H_{\mathrm{experi}–}L_{\mathrm{expres}–}M_{\mathrm{physio}}$	−0.32	−4.105 (4.062)	0.02	0.063 (0.680)	−0.08	−0.049 (0.208)
$L_{\mathrm{experi}–}L_{\mathrm{expres}–}L_{\mathrm{physio}}$	−0.57^x^	−7.134 (3.718)	−0.14	−0.267 (0.631)	−0.06	−0.036 (0.189)
Interaction (Reference groups: $H_{\mathrm{experi}–}H_{\mathrm{expres}–}H_{\mathrm{physio}}$ and Time 2)						
$H_{\mathrm{experi}–}H_{\mathrm{expres}–}L_{\mathrm{physio}}$ Time 1	0.12	1.454 (3.882)	0.12	0.237 (0.797)	0.30	0.177 (0.225)
$H_{\mathrm{experi}–}L_{\mathrm{expres}–}M_{\mathrm{physio}}$ Time 1	−0.13	−1.586 (3.889)	−0.42	−0.818 (0.805)	−0.02	−0.009 (0.227)
$L_{\mathrm{experi}–}L_{\mathrm{expres}–}L_{\mathrm{physio}}$ Time1	0.02	0.187 (3.585)	−0.11	−0.215 (0.748)	0.05	0.030 (0.210)
$H_{\mathrm{experi}–}H_{\mathrm{expres}–}L_{\mathrm{physio}}$ Time 3	0.71^x^	8.851 (4.526)	0.82^x^	1.593 (0.966)	0.08	0.049 (0.252)
$H_{\mathrm{experi}–}L_{\mathrm{expres}–}M_{\mathrm{physio}}$ Time 3	0.60^x^	7.516 (4.349)	0.66	1.286 (0.922)	0.18	0.105 (0.249)
$L_{\mathrm{experi}–}L_{\mathrm{expres}–}L_{\mathrm{physio}}$ Time 3	0.75*	9.390 (4.114)	0.63	1.230 (0.875)	−0.02	−0.011 (0.232)
N (observations)		255		255		270
N (individuals)		109		109		109
R2 (fixed)		0.217		0.140		0.049
R2 (total)		0.640		0.311		0.412

** *p* < 0.01.

* *p* < 0.05.

x *p* ≤ 0.10.

**Table 3.** Linear mixed effect regression model results demonstrating group differences in indices of well-being over time based on stress experience, expression, and physiology profile

	Positive Affect *B*, b (SE)		Global Self-worth *B*, b (SE)		Satisfaction with Life *B*, b (SE)	
Age (years)	−0.16	−1.269 (0.870)	−0.09	−0.073 (0.084)	−0.05	−0.420 (0.832)
Takes Medication Affecting HPA	−0.21*	−3.471 (1.512)	−0.24**	−0.410 (0.144)	−0.37***	−6.058 (1.466)
Above Median Income	−0.00	0.088 (1.469)	0.07	0.151 (0.142)	0.06	1.110 (1.335)
Time Point (Reference: Time 2)						
Time 1	−0.48	−3.951 (2.426)	−0.26	−0.221 (0.260)	0.06	0.463 (1.951)
Time 3	0.43	3.508 (2.993)	0.52	0.432 (0.299)	0.32	2.588 (2.311)
Profile (Reference:$H_{\mathrm{experi}-}H_{\mathrm{expres}-}H_{\mathrm{physio}}$ )						
$H_{\mathrm{experi}-}H_{\mathrm{expres}-}L_{\mathrm{physio}}$	−0.02	−0.197 (2.854)	−0.28	−0.233 (0.284)	−0.06	−0.469 (2.562)
$H_{\mathrm{experi}-}L_{\mathrm{expres}-}M_{\mathrm{physio}}$	−0.38	−3.128 (2.913)	−0.30	−0.250 (0.287)	0.10	0.782 (2.615)
$L_{\mathrm{experi}-}L_{\mathrm{expres}-}L_{\mathrm{physio}}$	0.14	1.178 (2.621)	0.40	0.330 (0.254)	0.60*	4.829 (2.343)
Interaction (Reference groups: $H_{\mathrm{experi}-}H_{\mathrm{expres}-}H_{\mathrm{physio}}$ and Time 2)						
$H_{\mathrm{experi}-}H_{\mathrm{expres}-}L_{\mathrm{physio}}$ Time 1	0.63^x^	5.134 (3.070)	0.06	0.074 (0.329)	−0.08	−0.614 (2.385)
$H_{\mathrm{experi}-}L_{\mathrm{expres}-}M_{\mathrm{physio}}$ Time 1	1.00**	8.162 (3.105)	0.77	0.396 (0.321)	−0.06	0.478 (2.464)
$L_{\mathrm{experi}-}L_{\mathrm{expres}-}L_{\mathrm{physio}}$ Time 1	0.58^x^	4.736 (2.826)	0.19	0.160 (0.292)	−0.18	−1.446 (2.213)
$H_{\mathrm{experi}-}H_{\mathrm{expres}-}L_{\mathrm{physio}}$ Time 3	−0.61	−5.014 (3.824)	−0.88^x^	−0.730 (0.378)	−0.23	−1.872 (2.929)
$H_{\mathrm{experi}-}L_{\mathrm{expres}-}M_{\mathrm{physio}}$ Time 3	−0.06	−0.459 (3.708)	−0.30	−0.250 (0.366)	−0.23	−1.832 (2.850)
$L_{\mathrm{experi}-}L_{\mathrm{expres}-}L_{\mathrm{physio}}$ Time 3	−0.62	−5.033 (3.380)	−0.68*	−0.566 (0.334)	−0.50*	−4.029 (2.581)
N (observations)		212		215		208
N (individuals)		98		101		98
R2 (fixed)		0.111		0.163		0.208
R2 (total)		0.514		0.525		0.709

*** *p* < 0.001.

** *p* < 0.01.

* *p* < 0.05.

x *p* ≤ 0.10.
