# Supplementary material for: Stress system concordance as a predictor of longitudinal patterns of resilience in adolescence
Source: Dev Psychopathol. Author manuscript; Available in PMC 2024 Jun 1. (PMC10784418; doi:10.1017/S0954579423000731)
Supplement: 1 [file NIHMS1909530-supplement-1.docx]

Stress system concordance as a predictor of longitudinal resilience trajectories in adolescence

SUPPLEMENTAL MATERIALS

[Table S1. Correlation matrix and item/ scale descriptives. 2](#_ty5l9qnapnbh)

[Table S2. Correlation matrix of profile attributes 4](#_y4i6z8okb2za)

[Table S3. Patterns and predictors of missing Beck Depression Inventory data across the four profiles. 5](#_ul5dntwbgnp9)

[Table S4. Patterns and predictors of missing Beck Suicide Severity Index data across the four profiles. 6](#_y8o5l9mkv4lc)

[Table S5. Patterns and predictors of missing non-suicidal self-injury data across the four profiles. 7](#_4z0rv5mc2cvn)

[Table S6. Patterns and predictors of missing Positive Affect data across the four profiles. 8](#_d9pc6jn7i0pt)

[Table S7. Patterns and predictors of missing Self Worth data across the four profiles. 9](#_un7cj44oclm7)

[Table S8. Patterns and predictors of missing Satisfaction with Life Scale data across the four profiles. 10](#_hoqpj0yoryyu)

[Table S9. Parameter Estimates (Standard Errors) and Model Adequacy Indices for Subgroups Produced by Multi-trajectory Models. 11](#_e1l1digkk59f)

[Table S10. History of Non-suicidal Self-injury by Profile 12](#_lqpw4obzkc6g)

[Table S11. Linear mixed effect regression model results demonstrating group differences in indices of psychopathology over time based on stress experience, expression, and physiology profile where Lexperi–Lexpres–Lphysio is set as the reference group. 13](#_90fszmvl1q9a)

[Table S12. Contrasts to detect differences in psychopathology between profiles at each time point. 14](#_zfk1h51a19j3)

[Table S13. Contrasts to detect significant change in symptoms of psychopathology across time points within each profile. 15](#_jqfhlvwqcaid)

[Table S14. Linear mixed effect regression model results demonstrating group differences in indices of well-being over time based on stress experience, expression, and physiology profile where Lexperi–Lexpres–Lphysio is set as the reference group. 16](#_vrys8r6j2irb)

[Table S15. Contrasts to detect differences in well-being between profiles at each time point. 17](#_myq132u7gm03)

[Table S16. Contrasts to detect significant change in well-being across time points within each profile. 18](#_8eu85qocnfda)

# **Table S1.** Correlation matrix and item/ scale descriptives.

|  |  | 1 | 2 | 3 | 4 | 5 | 6 | 7 | 8 | 9 | 10 | 11 | 12 | 13 | 14 | 15 | 16 | 17 | 18 | 19 | 20 | 21 | 22 |
| --- | --- | --- | --- | --- | --- | --- | --- | --- | --- | --- | --- | --- | --- | --- | --- | --- | --- | --- | --- | --- | --- | --- | --- |
| 1 | Age T1 | - |  |  |  |  |  |  |  |  |  |  |  |  |  |  |  |  |  |  |  |  |  |
| 2 | Income T1 | 0.108 | - |  |  |  |  |  |  |  |  |  |  |  |  |  |  |  |  |  |  |  |  |
| 3 | Race/Ethnicity | 0.018 | **-0.360** | - |  |  |  |  |  |  |  |  |  |  |  |  |  |  |  |  |  |  |  |
| 4 | Medication T1 | 0.113 | -0.123 | -0.027 | - |  |  |  |  |  |  |  |  |  |  |  |  |  |  |  |  |  |  |
| 5 | BDI T1 | **0.216** | -0.156 | 0.140 | **0.247** | - |  |  |  |  |  |  |  |  |  |  |  |  |  |  |  |  |  |
| 6 | BDI T2 | 0.040 | -0.191 | 0.070 | **0.435** | **0.591** | - |  |  |  |  |  |  |  |  |  |  |  |  |  |  |  |  |
| 7 | BDI T3 | -0.156 | -0.098 | -0.025 | **0.274** | **0.613** | **0.674** | - |  |  |  |  |  |  |  |  |  |  |  |  |  |  |  |
| 8 | BSSI T1 | 0.016 | -0.147 | 0.111 | **0.281** | **0.731** | **0.453** | **0.429** | - |  |  |  |  |  |  |  |  |  |  |  |  |  |  |
| 9 | BSSI T2 | -0.116 | -0.181 | 0.196 | **0.306** | 0.188 | **0.576** | **0.311** | 0.207 | - |  |  |  |  |  |  |  |  |  |  |  |  |  |
| 10 | BSSI T3 | 0.002 | -0.171 | 0.056 | 0.220 | 0.250 | **0.349** | **0.594** | **0.300** | **0.407** | - |  |  |  |  |  |  |  |  |  |  |  |  |
| 11 | NSSI Engagement T1 | **0.199** | -0.035 | 0.066 | **0.243** | **0.511** | **0.458** | **0.418** | **0.362** | **0.231** | **0.254** | - |  |  |  |  |  |  |  |  |  |  |  |
| 12 | NSSI Engagement T2 | -0.005 | -0.032 | -0.003 | 0.153 | **0.389** | **0.574** | **0.530** | **0.312** | **0.455** | **0.354** | **0.602** | - |  |  |  |  |  |  |  |  |  |  |
| 13 | NSSI Engagement T3 | -0.119 | 0.091 | -0.096 | 0.025 | 0.136 | **0.241** | **0.445** | 0.160 | **0.257** | **0.391** | 0.119 | **0.360** | - |  |  |  |  |  |  |  |  |  |
| 14 | Positive Affect T1 | -0.172 | 0.005 | -0.108 | **-0.256** | **-0.496** | **-0.312** | **-0.342** | **-0.390** | -0.206 | -0.228 | **-0.222** | **-0.299** | -0.167 | - |  |  |  |  |  |  |  |  |
| 15 | Positive Affect T2 | -0.163 | 0.146 | -0.150 | -0.105 | **-0.365** | **-0.391** | **-0.314** | **-0.301** | -0.218 | -0.128 | -0.221 | **-0.238** | **-0.343** | **0.328** | - |  |  |  |  |  |  |  |
| 16 | Positive Affect T3 | 0.013 | 0.087 | -0.052 | -0.234 | **-0.539** | **-0.435** | **-0.416** | **-0.391** | -0.153 | -0.172 | -0.239 | **-0.363** | -0.194 | **0.378** | **0.670** | - |  |  |  |  |  |  |
| 17 | Self Worth T1 | -0.117 | **0.234** | **-0.241** | **-0.224** | **-0.728** | **-0.602** | **-0.480** | **-0.703** | **-0.368** | -0.166 | **-0.497** | **-0.489** | -0.126 | **0.549** | **0.371** | **0.454** | - |  |  |  |  |  |
| 18 | Self Worth T2 | -0.073 | 0.086 | -0.127 | **-0.260** | **-0.432** | **-0.649** | **-0.397** | **-0.412** | **-0.424** | **-0.321** | **-0.304** | **-0.341** | **-0.320** | **0.396** | **0.570** | **0.550** | **0.608** | - |  |  |  |  |
| 19 | Self Worth T3 | 0.082 | -0.080 | 0.046 | -0.202 | **-0.528** | **-0.562** | **-0.701** | **-0.321** | -0.145 | -0.239 | **-0.297** | -0.252 | **-0.267** | 0.106 | **0.460** | **0.667** | **0.399** | **0.518** | - |  |  |  |
| 20 | Life Satisfaction T1 | -0.120 | 0.087 | -0.105 | **-0.403** | **-0.646** | **-0.642** | **-0.597** | **-0.546** | **-0.443** | **-0.422** | **-0.362** | **-0.440** | -0.242 | **0.520** | **0.331** | **0.492** | **0.715** | **0.582** | **0.510** | - |  |  |
| 21 | Life Satisfaction T2 | 0.016 | **0.254** | **-0.234** | **-0.385** | **-0.501** | **-0.745** | **-0.626** | **-0.465** | **-0.514** | **-0.319** | **-0.410** | **-0.448** | **-0.319** | **0.331** | **0.539** | **0.484** | **0.505** | **0.758** | **0.588** | **0.668** | - |  |
| 22 | Life Satisfaction T3 | -0.032 | 0.120 | -0.097 | **-0.353** | **-0.546** | **-0.578** | **-0.626** | **-0.305** | **-0.338** | **-0.395** | **-0.276** | **-0.368** | **-0.379** | **0.444** | **0.584** | **0.571** | **0.434** | **0.491** | **0.761** | **0.737** | **0.785** | - |
|  | N | 109 | 109 | 109 | 109 | 103 | 86 | 64 | 106 | 85 | 62 | 107 | 89 | 73 | 82 | 73 | 55 | 81 | 75 | 58 | 81 | 70 | 55 |
|  | Mean (SD) | *14.94 (1.20)* | *na* | *na* | *na* | 15.51 (14.16) | 14.15 (11.34) | 13.92 (10.75) | 1.42 (2.16) | 1.01 (1.69) | 0.89 (1.78) | 0.53 (0.63) | 0.42 (0.54) | 0.35 (0.56) | 25.87 (8.00) | 23.68 (8.00) | 23.65 (8.50) | 2.62 (0.89) | 2.62 (0.78) | 2.59 (0.85) | 22.67 (8.16) | 22.03 (7.92) | 22.18 (7.88) |
|  | Range | 12.20-17.02 | 0, 1 | 0, 1 | 0, 1 | 0–54 | 0–38 | 0–43 | 0–10 | 0–7 | 0–7 | 0–  2.53 | 0–  2.56 | 0–  1.96 | 10–43 | 10–46 | 10–45 | 1–4 | 1–4 | 1–4 | 5–35 | 5–35 | 5–35 |
|  | Skewness | *na* | *na* | *na* | *na* | 0.782 | 0.430 | 0.735 | 1.876 | 1.652 | 2.502 | 1.036 | 1.474 | 1.510 | 0.196 | 0.776 | 0.501 | -0.070 | -0.164 | -0.025 | -0.382 | -0.144 | -0.223 |
|  | Kurtosis | *na* | *na* | *na* | *na* | -0.331 | -1.017 | -0.030 | 3.661 | 1.895 | 5.896 | 0.484 | 2.167 | 1.088 | -0.506 | 0.282 | -0.296 | -0.919 | -0.701 | -0.877 | -0.790 | -1.040 | -0.955 |
|  | Cronbach’s alpha | *na* | *na* | *na* | *na* | 0.96 | 0.93 | 0.96 | 0.87 | 0.86 | 0.86 | *na* | *na* | *na* | 0.89 | 0.91 | 0.91 | 0.91 | 0.92 | 0.92 | 0.93 | 0.89 | 0.91 |

*Note:* Bold indicates significance at *p* < 0.05. Income levels were divided into high/low using a median split. Race/Ethnicity was divided into those with a minoritized identity and those without. Medication captures whether the individual was taking a medication that is known to act on the HPA axis or not. T1=Time 1, T2= Time 2, T3= Time 3, BDI=Beck Depression Inventory II Total Score, BSSI=Beck Suicide Severity Inventory 5-tem Score, NSSI=Non-suicidal self-injury (past year counts log transformed).

# **Table S2.** Correlation matrix of profile attributes

#

|  |  | 1 | 2 | 3 | 4 |
| --- | --- | --- | --- | --- | --- |
| 1 | Self-reported Stress, Speech | - |  |  |  |
| 2 | Self-reported Stress, Math | **0.476** | - |  |  |
| 3 | Experimenter Rated Stress, Speech | **0.212** | 0.0853 | - |  |
| 4 | Experimenter Reported Stress, Math | 0.053 | **0.385** | **0.512** | - |
| 5 | Mid-TSST Cortisol (Sample #3)* | 0.048 | 0.044 | 0.047 | 0.040 |

*Note:* Bold indicates significance at *p* < 0.05. * Cortisol Sample #3 was selected for the correlation table as this sample is time-lagged to index peak cortisol response during the Trier Social Stress Test (see Figure 1). TSST = Trier Social Stress Test

#

# **Table S3.** Patterns and predictors of missing Beck Depression Inventory data across the four profiles.

|  | LLL  (N = 39) | | HLM  (N = 25) | | HHH  (N = 19) | | HHL  (N = 26) | | ALL  (N = 109) | |
| --- | --- | --- | --- | --- | --- | --- | --- | --- | --- | --- |
| Beck Depression Inventory II Missingness, b (se) | | | | | | | | | | |
|  | T2 | T3 | T2 | T3 | T2 | T3 | T2 | T3 | T2 | T3 |
| *N* (sample with data) | 32 | 25 | 21 | 17 | 12 | 9 | 23 | 13 | 88 | 64 |
| Missing Data Correlates | | | | | | | | | | |
| Age | *ns* | *ns* | *ns* | 1.07, 0.60 | *ns* | *ns* | *ns* | *ns* | *ns* | *ns* |
| Income | *ns* | -1.40, 0.85 | *ns* | *ns* | *ns* | *ns* | *ns* | *ns* | *ns* | *ns* |
| Minority race | *ns* | *ns* | *ns* | *ns* | *ns* | *ns* | *ns* | *ns* | *ns* | *ns* |
| BDI | *ns* | *ns* | *ns* | *ns* | *ns* | *ns* | *ns* | 0.08, 0.04* | *ns* | 0.03, 0.01* |
| BSSI | *ns* | *ns* | *ns* | *ns* | *ns* | *ns* | *ns* | *ns* | *ns* | *ns* |
| NSSI | *ns* | *ns* | *ns* | *ns* | *ns* | *ns* | *ns* | *ns* | 0.74, 0.36* | *ns* |
| PA | *ns* | *ns* | *ns* | *ns* | *ns* | *ns* | *ns* | *ns* | *ns* | *ns* |
| SPPA | *ns* | *ns* | *ns* | *ns* | *ns* | *ns* | *ns* | *ns* | *ns* | -0.45, 0.27 |
| SLS | -0.22, 0.12 | *ns* | *ns* | *ns* | *ns* | *ns* | *ns* | *ns* | *ns* | *ns* |

*Note:* LLL = Low experience, Low expression, and Low physiology, HLM = High experience, Low expression, and Moderate physiology, HHH = High experience, High expression, and High physiology, HHL = High experience, High expression, and Low physiology, ALL = total study sample, T2 = Time 2, T3 = Time 3, BDI = Beck Depression Inventory II, BSSI = Beck Suicide Severity Index, NSSI = Non-suicidal self-injury, PA = Positive Affect subscale of the Positive and Negative Affect Schedule - Extended, SPPA = The Self-Perception Profile for Adolescents Global Self-worth subscale, SLS = Satisfaction with Life Scale.

Non-standard beta values and standard deviations are provided when *p* < 0.1, * indicates *p* < 0.05.

# **Table S4.** Patterns and predictors of missing Beck Suicide Severity Index data across the four profiles.

|  | LLL  (N = 39) | | HLM  (N = 25) | | HHH  (N = 19) | | HHL  (N = 26) | | ALL  (N = 109) | |
| --- | --- | --- | --- | --- | --- | --- | --- | --- | --- | --- |
| Beck Suicide Severity Index Missingness, b (se) | | | | | | | | | | |
|  | T2 | T3 | T2 | T3 | T2 | T3 | T2 | T3 | T2 | T3 |
| *N* (sample with data) | 32 | 24 | 21 | 17 | 12 | 9 | 23 | 12 | 88 | 62 |
| Missing Data Correlates | | | | | | | | | | |
| Age | 0.79, 0.39* | 0.53, 0.30 | *ns* | 1.07, 0.60 | *ns* | *ns* | *ns* | *ns* | *ns* | 0.32, 0.17 |
| Income | *ns* | *ns* | *ns* | *ns* | *ns* | *ns* | *ns* | *ns* | *ns* | *ns* |
| Minority race | *ns* | *ns* | *ns* | *ns* | *ns* | *ns* | *ns* | *ns* | *ns* | *ns* |
| BDI | *ns* | *ns* | *ns* | *ns* | *ns* | *ns* | *ns* | 0.09, 0.04* | *ns* | 0.04, 0.01* |
| BSSI | *ns* | *ns* | *ns* | *ns* | *ns* | *ns* | *ns* | *ns* | *ns* | *ns* |
| NSSI | *ns* | *ns* | *ns* | *ns* | *ns* | *ns* | *ns* | *ns* | 0.66, 0.36 | *ns* |
| PA | *ns* | *ns* | *ns* | *ns* | *ns* | *ns* | *ns* | *ns* | *ns* | -0.06, 0.03 |
| SPPA | *ns* | *ns* | *ns* | *ns* | *ns* | *ns* | *ns* | *ns* | *ns* | -0.46, 0.27 |
| SLS | *ns* | *ns* | *ns* | *ns* | *ns* | *ns* | *ns* | *ns* | *ns* | *ns* |

*Note:* LLL = Low experience, Low expression, and Low physiology, HLM = High experience, Low expression, and Moderate physiology, HHH = High experience, High expression, and High physiology, HHL = High experience, High expression, and Low physiology, ALL = total study sample, T2 = Time 2, T3 = Time 3, BDI = Beck Depression Inventory II, BSSI = Beck Suicide Severity Index, NSSI = Non-suicidal self-injury, PA = Positive Affect subscale of the Positive and Negative Affect Schedule - Extended, SPPA = The Self-Perception Profile for Adolescents Global Self-worth subscale, SLS = Satisfaction with Life Scale.

Non-standard beta values and standard deviations are provided when *p* < 0.1, * indicates *p* < 0.05.

# **Table S5.** Patterns and predictors of missing non-suicidal self-injury data across the four profiles.

|  | LLL  (N = 39) | | HLM  (N = 25) | | HHH  (N = 19) | | HHL  (N = 26) | | ALL  (N = 109) | |
| --- | --- | --- | --- | --- | --- | --- | --- | --- | --- | --- |
| Non-suicidal Self-injury Missingness, b (se) | | | | | | | | | | |
|  | **T2** | **T3** | **T2** | **T3** | **T2** | **T3** | **T2** | **T3** | **T2** | **T3** |
| *N* (sample with data) | 35 | 28 | 21 | 19 | 13 | 10 | 23 | 16 | 92 | 73 |
| Missing Data Correlates | | | | | | | | | | |
| Age | 0.89, 0.49 | *ns* | *ns* | *ns* | *ns* | *ns* | *ns* | *ns* | *ns* | *ns* |
| Income | -2.19, 1.12 | -2.02, 0.92* | *ns* | *ns* | *ns* | *ns* | *ns* | *ns* | *ns* | *ns* |
| Minority race | *ns* | *ns* | *ns* | *ns* | *ns* | *ns* | *ns* | *ns* | *ns* | *ns* |
| BDI | *ns* | *ns* | *ns* | *ns* | *ns* | *ns* | *ns* | *ns* | *ns* | 0.03, 0.01 |
| BSSI | *ns* | *ns* | 0.144, 0.08 | *ns* | *ns* | *ns* | *ns* | *ns* | *ns* | *ns* |
| NSSI | *ns* | *ns* | *ns* | *ns* | *ns* | *ns* | *ns* | *ns* | 0.87, 0.38* | *ns* |
| PA | *ns* | *ns* | *ns* | *ns* | 0.15, 0.09 | *ns* | *ns* | *ns* | *ns* | *ns* |
| SPPA | *ns* | *ns* | *ns* | *ns* | *ns* | *ns* | *ns* | -1.99, 1.10 | *ns* | -0.58, 0.29* |
| SLS | *ns* | *ns* | *ns* | *ns* | *ns* | *ns* | *ns* | *ns* | *ns* | *ns* |

*Note:* LLL = Low experience, Low expression, and Low physiology, HLM = High experience, Low expression, and Moderate physiology, HHH = High experience, High expression, and High physiology, HHL = High experience, High expression, and Low physiology, ALL = total study sample, T2 = Time 2, T3 = Time 3, BDI = Beck Depression Inventory II, BSSI = Beck Suicide Severity Index, NSSI = Non-suicidal self-injury, PA = Positive Affect subscale of the Positive and Negative Affect Schedule - Extended, SPPA = The Self-Perception Profile for Adolescents Global Self-worth subscale, SLS = Satisfaction with Life Scale.

Non-standard beta values and standard deviations are provided when *p* < 0.1, * indicates *p* < 0.05.

# **Table S6.** Patterns and predictors of missing Positive Affect data across the four profiles.

|  | LLL  (N = 39) | | HLM  (N = 25) | | HHH  (N = 19) | | HHL  (N = 26) | | ALL  (N = 109) | |
| --- | --- | --- | --- | --- | --- | --- | --- | --- | --- | --- |
| Positive Affect Scale Missingness, b (se) | | | | | | | | | | |
|  | **T2** | **T3** | **T2** | **T3** | **T2** | **T3** | **T2** | **T3** | **T2** | **T3** |
| *N* (sample with data) | 28 | 24 | 17 | 13 | 13 | 7 | 17 | 11 | 75 | 55 |
| Missing Data Correlates | | | | | | | | | | |
| Age | ns | ns | ns | ns | ns | ns | ns | 0.749, 0.424 | ns | 0.298,0.168 |
| Income | ns | ns | ns | ns | ns | ns | ns | ns | ns | -0.875, 0.499 |
| Minority race | ns | ns | ns | ns | ns | ns | ns | ns | ns | ns |
| BDI | ns | ns | ns | ns | ns | ns | ns | 0.109, 0.044* | 0.027, 0.015 | 0.035,0.015* |
| BSSI | ns | ns | 0.269, 0.128* | ns | ns | ns | ns | ns | 0.048, 0.026 | ns |
| NSSI | 2.279, 0.827* | ns | 1.702, 0.866* | ns | ns | ns | 2.183, 0.923* | ns | 1.747, 0.412* | ns |
| PA | ns | -0.155,0.080 | ns | ns | 0.154, 0.085 | ns | ns | ns | ns | ns |
| SPPA | ns | ns | ns | ns | ns | ns | -2.230, 1.245 | ns | -0.597, 0.288* | -0.675, 0.275* |
| SLS | ns | ns | ns | 0.149, 0.090 | ns | ns | ns | ns | ns | ns |

*Note:* LLL = Low experience, Low expression, and Low physiology, HLM = High experience, Low expression, and Moderate physiology, HHH = High experience, High expression, and High physiology, HHL = High experience, High expression, and Low physiology, ALL = total study sample, T2 = Time 2, T3 = Time 3, BDI = Beck Depression Inventory II, BSSI = Beck Suicide Severity Index, NSSI = Non-suicidal self-injury, PA = Positive Affect subscale of the Positive and Negative Affect Schedule - Extended, SPPA = The Self-Perception Profile for Adolescents Global Self-worth subscale, SLS = Satisfaction with Life Scale.

Non-standard beta values and standard deviations are provided when *p* < 0.1, * indicates *p* < 0.05.

# **Table S7.** Patterns and predictors of missing Self Worth data across the four profiles.

|  | LLL  (N = 39) | | HLM  (N = 25) | | HHH  (N = 19) | | HHL  (N = 26) | | ALL  (N = 109) | |
| --- | --- | --- | --- | --- | --- | --- | --- | --- | --- | --- |
| Self-Perception Profile for Adolescents Missingness, b (se) | | | | | | | | | | |
|  | **T2** | **T3** | **T2** | **T3** | **T2** | **T3** | **T2** | **T3** | **T2** | **T3** |
| *N* (sample with data) | 31 | 25 | 17 | 14 | 13 | 7 | 16 | 12 | 77 | 58 |
| Missing Data Correlates | | | | | | | | | | |
| Age | ns | ns | ns | 0.838, 0.491 | ns | ns | ns | ns | ns | 0.335, 0.171* |
| Income | ns | -1.467, 0.863 | ns | ns | ns | ns | ns | ns | ns | -0.933, 0.502 |
| Minority race | ns | ns | ns | ns | ns | ns | ns | ns | ns | ns |
| BDI | 0.074,0.034* | ns | ns | ns | ns | ns | ns | 0.094, 0.040* | 0.037, 0.015* | 0.033, 0.014* |
| BSSI | ns | ns | ns | ns | ns | ns | ns | ns | ns | ns |
| NSSI | 1.691, 0.711* | ns | ns | ns | ns | ns | 1.684, 0.788* | ns | 1.364, 0.376* | ns |
| PA | ns | ns | ns | ns | 0.154, 0.085 | ns | -0.194, 0.117 | ns | ns | -0.051, 0.029 |
| SPPA | -1.809,0.622 | ns | ns | ns | ns | ns | ns | ns | ns | -0.849, 0.289* |
| SLS | -0.158, 0.082 | ns | ns | ns | ns | ns | ns | ns | ns | ns |

*Note:* LLL = Low experience, Low expression, and Low physiology, HLM = High experience, Low expression, and Moderate physiology, HHH = High experience, High expression, and High physiology, HHL = High experience, High expression, and Low physiology, ALL = total study sample, T2 = Time 2, T3 = Time 3, BDI = Beck Depression Inventory II, BSSI = Beck Suicide Severity Index, NSSI = Non-suicidal self-injury, PA = Positive Affect subscale of the Positive and Negative Affect Schedule - Extended, SPPA = The Self-Perception Profile for Adolescents Global Self-worth subscale, SLS = Satisfaction with Life Scale.

Non-standard beta values and standard deviations are provided when *p* < 0.1, * indicates *p* < 0.05.

# **Table S8.** Patterns and predictors of missing Satisfaction with Life Scale data across the four profiles.

|  | LLL  (N = 39) | | HLM  (N = 25) | | HHH  (N = 19) | | HHL  (N = 26) | | ALL  (N = 109) | |
| --- | --- | --- | --- | --- | --- | --- | --- | --- | --- | --- |
| Satisfaction with Life Scale Missingness, b (se) | | | | | | | | | | |
|  | **T2** | **T3** | **T2** | **T3** | **T2** | **T3** | **T2** | **T3** | **T2** | **T3** |
| *N* (sample with data) | 27 | 24 | 16 | 13 | 13 | 7 | 16 | 11 | 72 | 55 |
| Missing Data Correlates | | | | | | | | | | |
| Age | ns | ns | ns | ns | ns | ns | ns | 0.749, 0.424 | ns | 0.298, 0.168 |
| Income | ns | ns | ns | ns | ns | ns | ns | ns | ns | -0.875, 0.499 |
| Minority race | ns | ns | ns | ns | ns | ns | ns | ns | ns | ns |
| BDI | ns | ns | ns | ns | ns | ns | ns | 0.109, 0.044* | ns | 0.036, 0.015* |
| BSSI | ns | ns | 0.233, 0.119* | ns | ns | ns | ns | ns | 0.046, 0.026 | ns |
| NSSI | 2.133, 0.795* | ns | 1.354, 0.811 | ns | ns | ns | 2.01, 0.866* | ns | 1.599, 0.396* | ns |
| PA | ns | -0.156, 0.080 | ns | ns | 0.154, 0.085 | ns | ns | ns | ns | ns |
| SPPA | ns | ns | ns | ns | ns | ns | -3.474, 1.709* | ns | -0.586, 0.280* | -0.675, 0.275* |
| SLS | ns | ns | ns | 0.149, 0.090 | ns | ns | ns | ns | ns | ns |

*Note:* LLL = Low experience, Low expression, and Low physiology, HLM = High experience, Low expression, and Moderate physiology, HHH = High experience, High expression, and High physiology, HHL = High experience, High expression, and Low physiology, ALL = total study sample, T2 = Time 2, T3 = Time 3, BDI = Beck Depression Inventory II, BSSI = Beck Suicide Severity Index, NSSI = Non-suicidal self-injury, PA = Positive Affect subscale of the Positive and Negative Affect Schedule - Extended, SPPA = The Self-Perception Profile for Adolescents Global Self-worth subscale, SLS = Satisfaction with Life Scale.

Non-standard beta values and standard deviations are provided when *p* < 0.1, * indicates *p* < 0.05.

# **Table S9.** Parameter Estimates (Standard Errors) and Model Adequacy Indices for Subgroups Produced by Multi-trajectory Models.

|  | | Stress Experience | Stress Expression | Salivary Cortisol | | | | | AvePP_j_ | | OCC_j_ | Prob_j_ | Prop_j_ | Ratio |
| --- | --- | --- | --- | --- | --- | --- | --- | --- | --- | --- | --- | --- | --- | --- |
| Low Experience - Low Expression - Low Physiology (n=41) | | | | | |  | | | .926 | | 37.379 | .359 | .362 | 0.992 |
| Intercept | 1.935* (0.158) ^A^ | | 2.238* (0.132) ^A^ | 0.101* (0.008) ^A^ | | | | | |  |  |  |  |  |
| Linear | 0.403* (0.049) | | 0.090* (0.037) ^a^ | -0.001^†^ (0.001) ^a^ | | | | |  | |  |  |  |  |
| Quadratic | -0.028* (0.003) ^a^ | |  |  | | | | |  | |  |  |  |  |
| High Experience – Low Expression – Moderate Physiology (n=25) | | | | | | | |  | .851 | | 17.263 | .219 | .221 | 0.991 |
| Intercept | 3.006* (0.228) ^B^ | | 1.908* (0.187) ^A^ | | 0.142* (0.014) ^B^ | | | | |  |  |  |  |  |
| Linear | 0.462* (0.064) | | 0.156* (0.049) ^a^ | 0.001* (0.001) | | | | |  | |  |  |  |  |
| Quadratic | -0.032* (0.004) ^a^ | |  | -0.001^†^ (0.001) ^b^ | | | | |  | |  |  |  |  |
| High Experience - High Expression - Low Physiology (n=28) | | | | | | |  | | .926 | | 37.724 | .252 | .248 | 1.016 |
| Intercept | 2.754* (0.197) ^B^ | | 2.897* (0.169) ^B^ | 0.090* (0.009) ^B^ | | | | | |  |  |  |  |  |
| Linear | 0.535* (0.057) | | 0.248* (0.047) ^b^ | -0.001^†^ (0.001) ^a^ | | | | |  | |  |  |  |  |
| Quadratic | -0.032* (0.003) ^a^ | |  |  | | | | |  | |  |  |  |  |
| High Experience - High Expression - High Physiology (n=19) | | | | | | |  | | .996 | | 828.898 | .169 | .168 | 1.006 |
| Intercept | 2.721* (0.212) ^B^ | | 3.018* (0.187) ^B^ | 0.223* (0.014) ^A^ | | | | |  | |  |  |  |  |
| Linear | 0.461* (0.067) | | 0.195* (0.052) ^b^ | 0.005* (0.001) | | | | |  | |  |  |  |  |
| Quadratic | -0.030* (0.004) ^a^ | |  | -0.001* (0.001) ^c^ | | | | |  | |  |  |  |  |

*Note:* AvePP_j_ = Average posterior probability; OCC_j_ = Odds of correct classification; Prob_j_ = Probability of group assignment; Prop_j_ = Proportion of children assigned to each group; Ratio = Ratio of Prob_j_ to Prop_j_; ^A, a, B, b, c^ Upper-case and lower-case superscripts denote significant differences in intercept and polynomial estimates, respectively, within the same Trier Social Stress Test response index.

^†^ *p* < .10 * *p* < .05.

# **Table S10.** History of Non-suicidal Self-injury by Profile

|  | Total Sample  (*N =* 109) | *L_experi–_L_expres–_*  *L_physio_* (*n=*39) | *H_experi–_H_expres–_H_physio_* (*n=*19) | *H_experi–_L_expres–_M_physio_* (*n=*25) | *H_experi–_H_expres–_*  *L_physio_* (*n=*26) |
| --- | --- | --- | --- | --- | --- |
| NSSI History, *N* (%) | 73 (67.0) | 24 (61.5) | 12 (63.2) | 16 (64.0) | 21 (80.8) |
| NSSI Incidents, *M* (SD) | 37.06 (79.99) | 36.23 (92.20) | 45.71 (102.93) | 31.32 (66.12) | 38.15 (56.06) |
| NSSI Severity, *N* (%) ^a^ |  |  |  |  |  |
| None | 35 (32.7) | 15 (38.5) | 6 (31.6) | 9 (36.0) | 5 (20.0) |
| Mild | 10 (9.3) | 4 (10.3) | 1 (5.3) | 4 (16.0) | 1 (4.0) |
| Moderate | 40 (37.4) | 12 (30.8) | 8 (42.1) | 7 (28.0) | 13 (52.0) |
| Severe | 22 (20.6) | 8 (20.5) | 3 (15.8) | 5 (20.0) | 6 (24.0) |

*Note:* L_experi–_L_expres–_L_physio_=Low experience, low expression, low physiology profile group; H_experi–_H_expres–_H_physio_=High experience, high expression, high physiology profile group; H_experi–_L_expres–_M_physio_=High experience, low expression, moderate physiology profile group; H_experi–_H_expres–_L_physio_=High experience, High expression, low physiology profile group; NSSI = non-suicidal self-injury; NSSI History = The number and percentage of individuals within each profile that report any history of NSSI; NSSI Incidents = The average and standard deviation of the lifetime number of engagements in NSSI reported by each profile; NSSI Severity = The number and percentage of individuals within each profile that meet each NSSI severity cut off. ^a^ Two individuals are missing lifetime NSSI categorization.

# **Table S11.** Linear mixed effect regression model results demonstrating group differences in indices of psychopathology over time based on stress experience, expression, and physiology profile where *L_experi–_L_expres–_L_physio_* is set as the reference group.

|  | Depressive Symptoms  *B,*  b (SE), *p-value* | | Suicide Ideation Severity  *B,* b (SE), *p-value* | | Non-suicidal Self-injury  *B,* b (SE), *p-value* | |
| --- | --- | --- | --- | --- | --- | --- |
| Age (years) | 0.15 | 1.861 (1.197), 0.123 | 0.02 | 0.048 (0.171), 0.780 | 0.08 | 0.049 (0.058), 0.397 |
| Takes Medication Affecting HPA | 0.22** | 5.684 (2.053), 0.007 | 0.23** | 0.937 (0.287), 0.002 | 0.13 | 0.154 (0.098), 0.120 |
| Income (Above Median) | -0.10 | -2.948 (1.897), 0.122 | -0.10 | -0.458 (0.304), 0.134 | -0.03 | -0.043 (0.099), 0.667 |
| Time Point (Reference: Time 2) |  |  |  |  |  |  |
| Time 1 | 0.22 | 2.720 (2.072), 0.191 | 0.23 | 0.451 (0.421), 0.285 | 0.17 | 0.099 (0.117), 0.400 |
| Time 3 | 0.10 | 1.207 (2.334), 0.606 | 0.01 | 0.012 (0.478), 0.981 | -0.26 | -0.150 (0.128), 0.242 |
| Profile (Reference: *L_experi–_L_expres–_L_physio_*) | |  |  |  |  |  |
| *H_experi–_H_expres–_L_physio_* | 0.86** | 10.740 (3.100), 0.001 | 0.32 | 0.634 (0.519), 0.223 | 0.07 | 0.040 (0.160), 0.801 |
| *H_experi–_L_expres–_M_physio_* | 0.24 | 3.030 (3.194), 0.344 | 0.17 | 0.330 (0.533), 0.537 | -0.02 | -0.013 (0.165), 0.935 |
| *H_experi–_H_expres–_H_physio_* | 0.57 | 7.134 (3.718), 0.056 | 0.14 | 0.267 (0.631), 0.673 | 0.06 | 0.036 (0.189), 0.851 |
| Interaction of Time Point and Profile  (Reference groups: *L_experi–_L_expres–_L_physio_* and Time 2) | | |  |  |  |  |
| *H_experi–_H_expres–_L_physio_* Time 1 | 0.10 | 1.267 (3.067), 0.680 | 0.23 | 0.452 (0.638), 0.480 | 0.25 | 0.147 (0.175), 0.403 |
| *H_experi–_L_expres–_M_physio_* Time 1 | -0.14 | -1.773 (3.072), 0.565 | -0.31 | -0.603 (0.645), 0.351 | -0.07 | -0.039 (0.177), 0.825 |
| *H_experi–_H_expres–_H_physio_* Time 1 | -0.02 | -0.187 (3.585), 0.958 | 0.11 | 0.215 (0.748), 0.774 | -0.05 | -0.030 (0.210), 0.886 |
| *H_experi–_H_expres–_L_physio_* Time 3 | -0.04 | -0.539 (3.558), 0.880 | 0.19 | 0.363 (0.766), 0.636 | 0.10 | 0.060 (0.196), 0.759 |
| *H_experi–_L_expres–_M_physio_* Time 3 | -0.15 | -1.874 (3.357), 0.578 | 0.03 | 0.056 (0.716), 0.938 | 0.20 | 0.116 (0.193), 0.550 |
| *H_experi–_H_expres–_H_physio_* Time 3 | -0.75* | -9.390 (4.114), 0.024 | -0.63 | -1.230 (0.875), 0.162 | 0.02 | 0.011 (0.232), 0.963 |
| N |  | 255 |  | 255 |  | 270 |
| N (grid) |  | 109 |  | 109 |  | 109 |
| R2 (fixed) |  | 0.217 |  | 0.140 |  | 0.049 |
| R2 (total) |  | 0.640 |  | 0.311 |  | 0.412 |

*** p* < 0.01*, * p* < 0.05

# **Table S12.** Contrasts to detect differences in psychopathology between profiles at each time point.

|  |  | Depressive Symptoms | | | Suicide Ideation Severity | | | Non-suicidal Self-injury | | |
| --- | --- | --- | --- | --- | --- | --- | --- | --- | --- | --- |
| Time Point | Contrast  (Reference: *H_experi–_H_expres–_H_physio_*) | *b* | SE | *p-value* | *b* | SE | *p-value* | *b* | SE | *p-value* |
| Time 1 | *H_experi–_H_expres–_L_physio_* | 5.06 | 3.57 | 0.275 | 0.605 | 0.565 | 0.462 | 0.182 | 0.183 | 0.508 |
| Time 2 | *H_experi–_H_expres–_L_physio_* | 3.61 | 3.95 | 0.561 | 0.368 | 0.664 | 0.791 | 0.005 | 0.202 | 0.999 |
| Time 3 | *H_experi–_H_expres–_L_physio_* | **12.46** | **4.54** | **0.013** | **1.961** | **0.815** | **0.032** | 0.054 | 0.229 | 0.949 |
| Time 1 | *H_experi–_L_expres–_M_physio_* | -5.69 | 3.67 | 0.218 | -0.755 | 0.580 | 0.331 | -0.058 | 0.187 | 0.920 |
| Time 2 | *H_experi–_L_expres–_M_physio_* | -4.10 | 4.07 | 0.500 | 0.063 | 0.681 | 0.989 | -0.049 | 0.208 | 0.949 |
| Time 3 | *H_experi–_L_expres–_M_physio_* | 3.41 | 4.44 | 0.655 | 1.350 | 0.767 | 0.145 | 0.056 | 0.228 | 0.946 |
| Time Point | Contrast  (Reference: *L_experi–_L_expres–_L_physio_*) | *b* | SE | *p-value* | *b* | SE | *p-value* | *b* | SE | *p-value* |
| Time 1 | *H_experi–_H_expres–_L_physio_* | **12.01** | **2.96** | **< .001** | **1.086** | **0.477** | **0.045** | 0.187 | 0.149 | 0.354 |
| Time 2 | *H_experi–_H_expres–_L_physio_* | **10.74** | **3.10** | **0.001** | 0.634 | 0.519 | 0.374 | 0.040 | 0.160 | 0.943 |
| Time 3 | *H_experi–_H_expres–_L_physio_* | **10.20** | **3.60** | **0.010** | 0.997 | 0.650 | 0.224 | 0.101 | 0.179 | 0.786 |
| Time 1 | *H_experi–_L_expres–_M_physio_* | 1.26 | 3.04 | 0.869 | -0.274 | 0.491 | 0.789 | -0.053 | 0.153 | 0.904 |
| Time 2 | *H_experi–_L_expres–_M_physio_* | 3.03 | 3.20 | 0.539 | 0.330 | 0.534 | 0.751 | -0.013 | 0.165 | 0.991 |
| Time 3 | *H_experi–_L_expres–_M_physio_* | 1.16 | 3.48 | 0.909 | 0.386 | 0.597 | 0.734 | 0.102 | 0.178 | 0.779 |

*Note: p-value* adjustment was executed using Dunnett’s method for multiple comparisons for two tests (each contrast per time). Bold text indicates *p* < 0.10.

# **Table S13.** Contrasts to detect significant change in symptoms of psychopathology across time points within each profile.

|  |  | Depressive Symptoms | | | Suicide Ideation Severity | | | Non-suicidal Self-injury | | |
| --- | --- | --- | --- | --- | --- | --- | --- | --- | --- | --- |
| Profile | Contrast | *b* | SE | *p-value* | *b* | SE | *p-value* | *b* | SE | *p-value* |
| *H_experi–_H_expres–_H_physio_* | Time 1 - Time 2 | 2.533 | 3.20 | 0.640 | 0.666 | 0.646 | 0.487 | 0.068 | 0.186 | 0.893 |
|  | Time 3 - Time 2 | **-8.183** | **3.67** | **0.052** | -1.219 | 0.761 | 0.199 | -0.140 | 0.205 | 0.711 |
|  | Time 3 - Time 1 | -3.319 | 3.37 | 0.514 | **-1.884** | **0.763** | **0.027** | -0.208 | 0.220 | 0.541 |
| *L_experi–_L_expres–_L_physio_* | Time 1 - Time 2 | 2.720 | 2.07 | 0.326 | 0.451 | 0.421 | 0.462 | 0.099 | 0.117 | 0.606 |
|  | Time 3 - Time 2 | 1.207 | 2.34 | 0.813 | 0.012 | 0.479 | 0.999 | -0.150 | 0.128 | 0.401 |
|  | Time 3 - Time 1 | -1.513 | 2.75 | 0.793 | -0.440 | 0.508 | 0.592 | -0.249 | 0.146 | 0.160 |
| *H_experi–_L_expres–_M_physio_* | Time 1 - Time 2 | 0.947 | 2.58 | 0.893 | -0.152 | 0.518 | 0.926 | 0.059 | 0.145 | 0.872 |
|  | Time 3 - Time 2 | -0.666 | 2.80 | 0.948 | 0.068 | 0.568 | 0.984 | -0.035 | 0.159 | 0.955 |
|  | Time 3 - Time 1 | -1.614 | 3.27 | 0.826 | 0.220 | 0.602 | 0.894 | -0.094 | 0.174 | 0.798 |
| *H_experi–_H_expres–_L_physio_* | Time 1 - Time 2 | 3.987 | 2.61 | 0.226 | 0.903 | 0.514 | 0.147 | 0.245 | 0.144 | 0.164 |
|  | Time 3 - Time 2 | 0.668 | 2.95 | 0.952 | 0.375 | 0.620 | 0.760 | -0.090 | 0.161 | 0.787 |
|  | Time 3 - Time 1 | -3.319 | 3.37 | 0.514 | -0.528 | 0.649 | 0.626 | -0.335 | 0.176 | 0.108 |

*Note: p-value* adjustment was executed using Dunnett’s method for multiple comparisons for two tests (each contrast per profile). Bold text indicates *p* < 0.10.

# **Table S14.** Linear mixed effect regression model results demonstrating group differences in indices of well-being over time based on stress experience, expression, and physiology profile where *L_experi–_L_expres–_L_physio_* is set as the reference group.

|  | Positive Affect  *B,* b (SE), *p-value* | | Global Self-worth  *B,* b (SE), *p-value* | | Satisfaction with Life  *B,* b (SE), *p-value* | |
| --- | --- | --- | --- | --- | --- | --- |
| Age (years) | -0.16 | -1.269 (0.870), 0.148 | -0.09 | -0.073 (0.084), 0.384 | -0.05 | -0.420 (0.832), 0.614 |
| Takes Medication Affecting HPA | -0.21* | -3.471 (1.512), 0.024 | -0.24** | -0.410 (0.144), 0.005 | -0.37*** | -6.058 (1.466), 0.000 |
| Income (Above Median) | 0.00 | 0.088 (1.469), 0.952 | 0.07 | 0.151 (0.142), 0.290 | 0.06 | 1.110 (1.335), 0.407 |
| Time Point (Reference: Time 2) |  |  |  |  |  |  |
| Time 1 | 0.10 | 0.785 (1.739), 0.652 | -0.07 | -0.061 (0.162), 0.707 | -0.12 | -0.983 (1.389), 0.480 |
| Time 3 | -0.19 | -1.525 (1.869), 0.416 | -0.16 | -0.134 (0.179), 0.455 | -0.18 | -1.441 (1.497), 0.337 |
| Profile (Reference: *L_experi–_L_expres–_L_physio_*) | |  |  |  |  |  |
| *H_experi–_H_expres–_L_physio_* | -0.17 | -1.376 (2.419), 0.570 | -0.68* | -0.563 (0.237), 0.018 | -0.66* | -5.299 (2.193), 0.017 |
| *H_experi–_L_expres–_M_physio_* | -0.53 | -4.306 (2.464), 0.082 | -0.70* | -0.581 (0.236), 0.015 | -0.51 | -4.047 (2.228), 0.071 |
| *H_experi–_H_expres–_H_physio_* | -0.14 | -1.178 (2.621), 0.654 | -0.40 | -0.330 (0.254), 0.195 | -0.60* | -4.829 (2.343), 0.041 |
| Interaction (Reference groups: *L_experi–_L_expres–_L_physio_* and Time 2) | | |  |  |  |  |
| *H_experi–_H_expres–_L_physio_* Time 1 | 0.05 | 0.398 (2.583), 0.878 | -0.10 | -0.086 (0.267), 0.748 | 0.10 | 0.833 (1.969), 0.673 |
| *H_experi–_L_expres–_M_physio_* Time 1 | 0.42 | 3.426 (2.623), 0.194 | 0.28 | 0.236 (0.255), 0.357 | 0.24 | 1.925 (2.062), 0.353 |
| *H_experi–_H_expres–_H_physio_* Time 1 | -0.58 | -4.736 (2.826), 0.096 | -0.19 | -0.160 (0.292), 0.585 | 0.18 | 1.446 (2.213), 0.515 |
| *H_experi–_H_expres–_L_physio_* Time 3 | 0.00 | 0.019 (3.026), 0.995 | -0.20 | -0.164 (0.292), 0.576 | 0.27 | 2.157 (2.342), 0.359 |
| *H_experi–_L_expres–_M_physio_* Time 3 | 0.56 | 4.574 (2.915), 0.119 | 0.38 | 0.315 (0.279), 0.261 | 0.27 | 2.196 (2.270), 0.335 |
| *H_experi–_H_expres–_H_physio_* Time 3 | 0.62 | 5.033 (3.380), 0.139 | 0.68 | 0.566 (0.334), 0.093 | 0.50 | 4.029 (2.581), 0.121 |
| N |  | 212 |  | 215 |  | 208 |
| N (grid) |  | 98 |  | 101 |  | 98 |
| R2 (fixed) |  | 0.111 |  | 0.163 |  | 0.208 |
| R2 (total) |  | 0.514 |  | 0.525 |  | 0.709 |

**** p* < 0.001*, ** p* < 0.01*, * p* < 0.05

# **Table S15.** Contrasts to detect differences in well-being between profiles at each time point.

|  |  | Positive Affect | | | Global Self-worth | | | Satisfaction with Life | | |
| --- | --- | --- | --- | --- | --- | --- | --- | --- | --- | --- |
| Time Point | Contrast  (Reference: *H_experi–_H_expres–_H_physio_*) | *b* | SE | *p-value* | *b* | SE | *p-value* | *b* | SE | *p-value* |
| Time 1 | *H_experi–_H_expres–_L_physio_* | 4.936 | 2.67 | 0.121 | -0.159 | 0.285 | 0.788 | -1.083 | 2.42 | 0.852 |
| Time 2 | *H_experi–_H_expres–_L_physio_* | -0.197 | 2.86 | 0.994 | -0.233 | 0.285 | 0.623 | -0.469 | 2.56 | 0.967 |
| Time 3 | *H_experi–_H_expres–_L_physio_* | -5.212 | 3.59 | 0.260 | **-0.963** | **0.351** | **0.013** | -2.341 | 3.05 | 0.657 |
| Time 1 | *H_experi–_L_expres–_M_physio_* | 5.035 | 2.70 | 0.118 | 0.145 | 0.277 | 0.807 | 1.261 | 2.48 | 0.817 |
| Time 2 | *H_experi–_L_expres–_M_physio_* | -3.128 | 2.92 | 0.461 | -0.250 | 0.287 | 0.588 | 0.782 | 2.62 | 0.924 |
| Time 3 | *H_experi–_L_expres–_M_physio_* | -3.587 | 3.55 | 0.499 | -0.501 | 0.352 | 0.271 | -1.050 | 3.04 | 0.903 |
| Time Point | Contrast  (Reference: *L_experi–_L_expres–_L_physio_*) | *b* | SE | *p-value* | *b* | SE | *p-value* | *b* | SE | *p-value* |
| Time 1 | *H_experi–_H_expres–_L_physio_* | -0.978 | 2.33 | 0.866 | **-0.649** | **0.232** | **0.011** | **-4.47** | **2.08** | **0.063** |
| Time 2 | *H_experi–_H_expres–_L_physio_* | -1.376 | 2.42 | 0.782 | **-0.563** | **0.237** | **0.036** | **-5.30** | **2.20** | **0.033** |
| Time 3 | *H_experi–_H_expres–_L_physio_* | -1.357 | 2.75 | 0.826 | **-0.727** | **0.263** | **0.012** | -3.14 | 2.38 | 0.323 |
| Time 1 | *H_experi–_L_expres–_M_physio_* | -0.880 | 2.35 | 0.889 | -0.345 | 0.222 | 0.217 | -2.12 | 2.13 | 0.509 |
| Time 2 | *H_experi–_L_expres–_M_physio_* | -4.306 | 2.47 | 0.151 | **-0.581** | **0.236** | **0.029** | -4.05 | 2.23 | 0.131 |
| Time 3 | *H_experi–_L_expres–_M_physio_* | 0.268 | 2.70 | 0.988 | -0.265 | 0.261 | 0.496 | -1.85 | 2.37 | 0.648 |

*Note: p-value* adjustment was executed using Dunnett’s method for multiple comparisons for two tests (each contrast per time). Bold text indicates *p* < 0.10.

# **Table S16.** Contrasts to detect significant change in well-being across time points within each profile.

|  |  | Positive Affect | | | Global Self-worth | | | Satisfaction with Life | | |
| --- | --- | --- | --- | --- | --- | --- | --- | --- | --- | --- |
| Profile | Contrast | *b* | SE | *p-value* | *b* | SE | *p-value* | *b* | SE | *p-value* |
| *H_experi–_H_expres–_H_physio_* | Time 1 - Time 2 | -3.951 | 2.43 | 0.191 | -0.221 | 0.261 | 0.605 | 0.463 | 1.96 | 0.949 |
|  | Time 3 - Time 2 | 3.508 | 3.00 | 0.404 | 0.432 | 0.300 | 0.265 | 2.588 | 2.32 | 0.434 |
|  | Time 3 - Time 1 | **7.459** | **3.31** | **0.049** | 0.653 | 0.352 | 0.120 | 2.124 | 2.68 | 0.640 |
| *L_experi–_L_expres–_L_physio_* | Time 1 - Time 2 | 0.785 | 1.74 | 0.850 | -0.061 | 0.162 | 0.888 | -0.983 | 1.39 | 0.696 |
|  | Time 3 - Time 2 | -1.525 | 1.87 | 0.626 | -0.134 | 0.179 | 0.669 | -1.441 | 1.50 | 0.531 |
|  | Time 3 - Time 1 | -2.311 | 2.17 | 0.466 | -0.073 | 0.205 | 0.899 | -0.458 | 1.84 | 0.944 |
| *H_experi–_L_expres–_M_physio_* | Time 1 - Time 2 | 4.212 | 2.19 | 0.105 | 0.175 | 0.215 | 0.625 | 0.942 | 1.79 | 0.807 |
|  | Time 3 - Time 2 | 3.049 | 2.45 | 0.361 | 0.182 | 0.236 | 0.655 | 0.755 | 1.98 | 0.885 |
|  | Time 3 - Time 1 | -1.163 | 2.71 | 0.861 | 0.007 | 0.264 | 0.999 | -0.186 | 2.27 | 0.991 |
| *H_experi–_H_expres–_L_physio_* | Time 1 - Time 2 | 1.183 | 2.15 | 0.793 | -0.147 | 0.229 | 0.737 | -0.150 | 1.67 | 0.990 |
|  | Time 3 - Time 2 | -1.506 | 2.52 | 0.764 | -0.298 | 0.245 | 0.378 | 0.716 | 1.98 | 0.896 |
|  | Time 3 - Time 1 | -2.689 | 2.69 | 0.506 | -0.151 | 0.273 | 0.792 | 0.866 | 2.19 | 0.879 |

*Note: p-value* adjustment was executed using Dunnett’s method for multiple comparisons for two tests (each contrast per profile). Bold text indicates *p* < 0.10.
